# Probing Substrate Transport Effects on Enzymatic Hydrogen Catalysis: An Alternative Proton Transfer Pathway in Putatively Sensory [FeFe] Hydrogenase

**DOI:** 10.1021/acscatal.3c02314

**Published:** 2023-07-26

**Authors:** Princess R. Cabotaje, Kaija Walter, Afridi Zamader, Ping Huang, Felix Ho, Henrik Land, Moritz Senger, Gustav Berggren

**Affiliations:** Molecular Biomimetics, Department of Chemistry, Ångström Laboratory, Uppsala University, Box 523, SE-75120 Uppsala, Sweden

**Keywords:** hydrogenases, hydrogen, substrate transport, redox catalysis, bioinorganic chemistry, enzyme catalysis

## Abstract

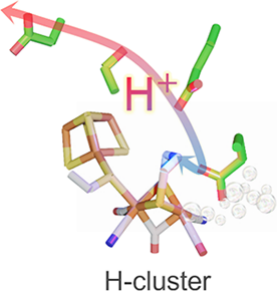

[FeFe] hydrogenases, metalloenzymes catalyzing proton/dihydrogen interconversion, have attracted intense attention due to their remarkable catalytic properties and (bio-)technological potential for a future hydrogen economy. In order to unravel the factors enabling their efficient catalysis, both their unique organometallic cofactors and protein structural features, i.e., “outer-coordination sphere” effects have been intensively studied. These structurally diverse enzymes are divided into distinct phylogenetic groups, denoted as Group A–D. Prototypical Group A hydrogenases display high turnover rates (10^4^–10^5^ s^–1^). Conversely, the sole characterized Group D representative, *Thermoanaerobacter mathranii* HydS (*Tam*HydS), shows relatively low catalytic activity (specific activity 10^–1^ μmol H_2_ mg^–1^ min^–1^) and has been proposed to serve a H_2_-sensory function. The various groups of [FeFe] hydrogenase share the same catalytic cofactor, the H-cluster, and the structural factors causing the diverging reactivities of Group A and D remain to be elucidated. In the case of the highly active Group A enzymes, a well-defined proton transfer pathway (PTP) has been identified, which shuttles H^+^ between the enzyme surface and the active site. In Group D hydrogenases, this conserved pathway is absent. Here, we report on the identification of highly conserved amino acid residues in Group D hydrogenases that constitute a possible alternative PTP. We varied two proposed key amino acid residues of this pathway (E252 and E289, *Tam*HydS numbering) via site-directed mutagenesis and analyzed the resulting variants via biochemical and spectroscopic methods. All variants displayed significantly decreased H_2_-evolution and -oxidation activities. Additionally, the variants showed two redox states that were not characterized previously. These findings provide initial evidence that these amino acid residues are central to the putative PTP of Group D [FeFe] hydrogenase. Since the identified residues are highly conserved in Group D exclusively, our results support the notion that the PTP is not universal for different phylogenetic groups in [FeFe] hydrogenases.

## Introduction

**Figure 1:**
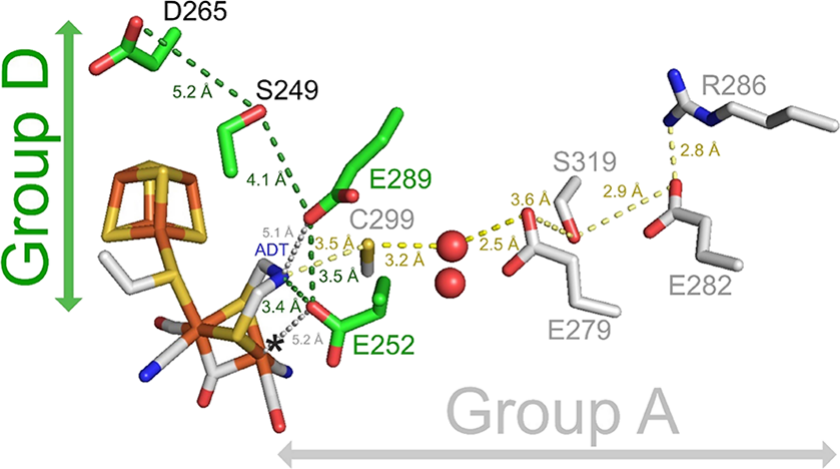
Overview of the proposed proton transfer pathway (PTP) in Group D [FeFe] hydrogenase *Tam*HydS (green) based on the YASARA-generated homology model^9^ versus the PTP of Group A representative, *Cp*I (gray; pdb: 4XDC).^10^ The iron in the H-cluster is colored in orange and the ADT bridgehead in blue. The apical open coordination site at the distal iron (Fe_d_) is indicated by an *. Residues belonging to the PTP of *Cp*I are presented as sticks in gray with water molecules (red spheres) in between C299 and E279; while that of *Tam*HydS are presented in green. In this study, the amino acids E252 and E289 are exchanged conservatively (E252D, E289D) and nonconservatively (E252V, E289A). The distance between ADT and E252 as well as between the side chains that are part of the proposed PTP of *Tam*HydS is numbered (dark green). The distances between Fe_d_ to E252 and ADT to E289 are indicated in gray dashed lines and labels. The yellow dashed lines and numbers indicate the distances between neighboring positions of the PTP of *Cp*I.^11^ The *Tam*HydS model does not include intraprotein waters which can likely compensate for the long distances between E289, S249, and D265.

[FeFe] hydrogenases are metalloenzymes that catalyze the reversible interconversion of electrons and protons to dihydrogen (H_2_), with turnover frequencies up to 10^5^ s^–1^.^1^ Due to their remarkable catalytic properties, these enzymes have been extensively studied and they inspire the design of new catalysts for H_2_ production and activation.^1a,2^ The reaction occurs at an iron–sulfur cofactor denoted the H-cluster, which consists of a [4Fe–4S] cluster connected via a bridging cysteine thiol to a di-iron ([2Fe]_H_) subsite. The Fe ions of the di-iron site, denoted as proximal (Fe_p_) and distal (Fe_d_) based on their location relative to the [4Fe–4S] cluster, are coordinated by CO and CN^–^ ligands, as well as a bridging azadithiolate ligand (^−^SCH_2_NCH_2_S^–^, ADT). The catalytic efficiency of the H-cluster is partly attributable to the asymmetric ligation of the [2Fe]_H_ subsite, which modulates the electronic structure across the metals and the ligands.^3^ Still, the exact nature of the [2Fe]_H_ subsite ligand-set is not crucial for reversible catalysis,^4^ and through the combined efforts of bio- and synthetic chemistry, it has become evident that the activity of the H-cluster is largely governed by factors beyond the primary coordination sphere.^5^ A striking example of the latter is provided by the amine group of the ADT ligand, which is critical for the shuttling of protons to the apical open coordination site at Fe_d_ (indicated by * in Figure 1), the site of H_2_ catalysis. The ADT-amine also serves as a base during H_2_ oxidation and can be regarded as forming a frustrated Lewis pair (FLP) with the Fe_d_ ion enabling heterolytic H_2_ cleavage. The role of the protein environment has also become evident through studies of semisynthetic [FeFe] hydrogenases. Indeed, incorporation of otherwise inactive synthetic di-iron complexes into the hydrogenase active-site pocket has been shown to dramatically alter their reactivity.^4,6^ Analogously, bioinspired design of synthetic catalysts, incorporating noncoordinating proton relays or embedding catalysts into larger polymer frameworks, has been shown to significantly enhance their catalytic properties.^7^ Elucidating and optimizing these second and outer coordination sphere effects is arguably the key to understanding hydrogenases and the development of the next generation of synthetic systems for hydrogen catalysis.^8^

[FeFe] hydrogenases are a diverse enzyme family, reflecting their broad range of functions related to hydrogen metabolism. Various classification schemes exist, but four phylogenetically distinct groups have been identified to date and denoted Group A–D.^1b,12^ The “prototypical” Group A [FeFe] hydrogenases are by far the most studied. Representative examples, including e.g., *Chlamydomonas reinhardtii* HydA1 (*Cr*HydA1),^13^*Desulfovibrio desulfuricans* HydAB (*Dd*HydAB),^14^ and *Clostridium pasteurianum* and *C. acetobutylicum* hydrogenase I (*Cp*I and *Ca*I),^15^ are the most active [FeFe] hydrogenases identified to date. While many Group A and B [FeFe] hydrogenases are involved in H_2_ production, the less studied Group C enzymes are considered sensory due to the presence of a signal-transducing Per-Arnt-Sim (PAS) domain.^16^ Finally, the Group D [FeFe] hydrogenases are most closely related to Group C but lack the PAS domain.^12a,16a^ Nevertheless, with the recent characterization of *Thermoanaerobacter mathranii* (*Tam*HydS) as a representative, Group D enzymes are also classified as putatively sensory.^9^

Data on Group C and D [FeFe] hydrogenases remain limited, but the representative examples characterized to date display properties clearly distinct from previously studied “prototypical” Group A enzymes.^9,16b^ The primary model systems for these nonprototypical [FeFe] hydrogenases include the Group C enzyme from *Thermotoga maritima* (*Tm*HydS)^16b,17^ and the aforementioned Group D enzyme *Tam*HydS. In particular, the reactivity of *Tam*HydS diverges from that of other [FeFe] hydrogenases with its low H_2_ evolution rate and a lower *K*_m_ for H_2_ (*K*_m_ 90–100 μM) than Group A and Group C enzymes (*K*_m_ > 400 μM).^9,16b,18^ Moreover, *Tam*HydS displays an unusual property of being a bidirectional catalyst, but with significant overpotential requirements for both H_2_ oxidation and proton reduction.^9,19^

As all [FeFe] hydrogenases share the same H-cluster, the varied reactivity of the enzymes must be due to changes in the surrounding protein environment.^12a,20^ In Group A enzymes, most modifications of the conserved amino acids surrounding the active site result in loss of function and in many cases also an inability to form the H-cluster.^21^ However, the phylogenetic groups vary in terms of their active-site pockets, which is expected to modulate the hydrogen bonding network and electrostatics around the H-cluster. The [FeFe] hydrogenases of different phylogenetic groups also differ in their electron and substrate (H^+^) transport pathways.^9,16b^ [FeFe] hydrogenases commonly feature additional FeS clusters (“F-clusters”) responsible for electron transfer during catalysis. These accessory cofactors have been shown to tune the reactivity of the H-cluster.^22^ However, they do not seem to be the key determinant of group-specific reactivity, as the FeS cluster content and location vary both across and within phylogenetic groups.^12a^ In addition to rapid electron transfer, the activity of [FeFe] hydrogenase is dependent on efficient proton transfer via PTPs. Alterations of the proton transfer kinetics have been shown to change the catalytic properties in Group A enzymes^11,23^ and could potentially explain the activity variations between different groups.

Examples of enzymes where phylogenetic groups feature different PTPs have been previously reported. The O_2_-processing heme–copper oxidase (HCuO) superfamily represents one of the most well-examined examples. The variations in PTPs observed across HCuO groups have been shown to correlate with differences in biochemical properties such as O_2_-affinity and proton pumping stoichiometry.^24^ These observations support the notion that the distinct reactivity of various phylogenetic groups of hydrogenases can be partly attributable to variations of their PTPs. Still, the nature and influence of different PTPs remain an open question.

The PTP(s) of [NiFe] hydrogenases remain to be firmly established.^25^ In the case of [FeFe] hydrogenase, the PTP of Group A enzymes has been studied extensively in recent years and shown to be composed of conserved amino acid residues and water molecules forming a hydrogen bonding network that connects the enzyme surface to the amine of the ADT ligand of the H-cluster (Figure 1).^11,23b,26^ The first amino acid of this PTP, C299 (*Cp*I numbering), is located within the H-bonding distance to the amine of the ADT ligand. Thus, in addition to proton transfer, C299_*Cp*I_ arguably influences the positioning of the amine base for efficient heterolytic H_2_ cleavage. Despite its seemingly critical importance for function, it is not conserved in Group C and Group D [FeFe] hydrogenases.^9,16b,17^ Instead, C299_*Cp*I_ is exchanged mostly into a hydrophobic alanine (e.g., Group C A131_*Tm*HydS,_ Group D A137_*Tam*HydS_). Restoring the proton transfer pathway (PTP) of Group A by exchanging A131_*Tm*HydS_ into a cysteine was attempted, but the variant lowered the capacity of Group C *Tm*HydS to oxidize and evolve H_2._^17^ However, beyond the aforementioned cysteine, the other residues crucial for proton transfer in Group A are also mostly exchanged into nonprotonatable hydrophobic side chains in *Tm*HydS. Similarly, in Group D *Tam*HydS, except for a conserved glutamate residue (E282_*Cp*I_, E122_*Tam*HydS_) and a conservative exchange of an arginine into a lysine (R286_*Cp*I_, K126_*Tam*HydS_), the other residues for proton transfer in Group A are missing (Figure 1). Since the buried H-cluster requires protons to be delivered to and from the bulk solvent to be active, this raises the question of alternative pathways, especially in sensory (HydS) [FeFe] hydrogenases.

**Figure 2:**
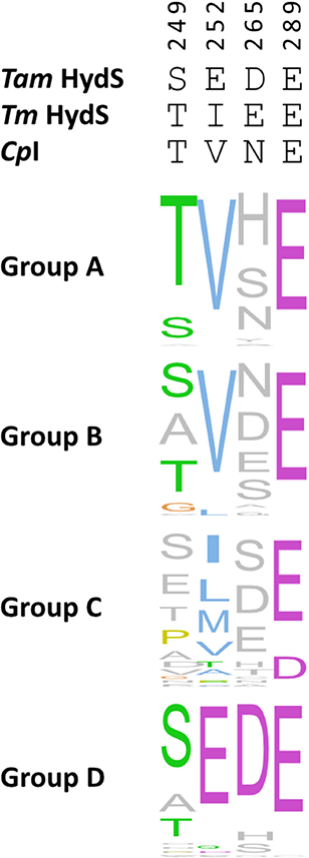
Normalized consensus logos of Groups A–D [FeFe] hydrogenase generated in Jalview using ClustalΩ sequence alignment^29^ of sequences retrieved from Greening et al.^30^ The numbering is based on the sequence of *Tam*HydS. The larger the font of the amino acid, the more conserved it is in the specified sequence position.

In this study, we examined the PTP of the putatively sensory Group D [FeFe] hydrogenase *Tam*HydS. Key amino acids with the highest potential to be part of the PTP were identified via homology modeling and alignment. Furthermore, our phylogenetic analysis of multiple Group D sequences showed that the identified amino acids are highly conserved in Group D [FeFe] hydrogenases (Figure 2). Site-directed mutagenesis of *Tam*HydS was used to target two proposed key amino acids (*Tam*HydS numbering): (a) E252, the residue with the highest potential to H bond with ADT according to distance and (b) E289, the most probable proton transferring neighbor to E252 (Figure 1). These two residues were individually substituted nonconservatively into a valine (E252V), alanine (E289A), and conservatively into an aspartate (E252D and E289D). Due to their proximity to the H-cluster, the selected amino acids also provide a probe for exploring how the outer-coordination sphere can alter the electronic distribution and geometry of the [2Fe]_H_ subsite. The catalytic rates of the variants were determined through biochemical assays and were shown to decrease relative to the wild-type enzyme in all variants. In parallel, attenuated total reflectance Fourier-transform infrared (ATR-FTIR) and electron paramagnetic resonance (EPR) spectroscopy were employed to probe the H-cluster. Our results demonstrate that variation of residues E252 and E289 causes accumulation of two distinct H-cluster states that were previously not characterized and that these residues play a crucial role for H_2_ catalysis in *Tam*HydS. The identified amino acids are likely to constitute the PTP in Group D and underscore that the proton transfer pathway is not universal in [FeFe] hydrogenases.

## Materials and Methods

### General

All chemicals were purchased from Sigma-Aldrich or VWR. Protein expression was analyzed by SDS-PAGE. All anaerobic work was performed in an MBRAUN glovebox ([O_2_] < 10 ppm). The synthetic cofactor [Fe_2_(μ-ADT)(CO)_4_(CN)_2_](Et_4_N)_2_ ([2Fe]^ADT^) was synthesized following literature protocols with minor modifications and verified by FTIR spectroscopy.^27^ UV–vis spectra were obtained using an AvaSpec-ULS2048-USB2-UA-50: Avantes Fiber Optic UV/VIS/NIR.

### In Silico Work and Sequence Alignment Analysis

The YASARA-generated homology model^9^ of Group D *Tam*HydS was aligned with the crystal structure of Group A representative, *Cp*I (pdb: 4XDC),^10^ using the MUSTANG^28^ multiple structural alignment algorithm in YASARA (Figure S1). Amino acid residues that could be part of the PTP were screened by checking all residues with H-bonding capacity around 5 Å, starting from the ADT of the H-cluster to the surface of the protein. From S249, the cut-off was extended up to 5.3 Å since there were no protonatable, polar, or electrically charged side chain within 5 Å. Amino acid sequence comparison of Group A–D in Figure 2 was based on a ClustalΩ sequence alignment^29^ of sequences retrieved from Greening et al.^30^ Normalized consensus logos of [FeFe] hydrogenase Groups A–D were generated in Jalview using the sequence alignment. The interatomic distances between the distal iron (Fe_d_) and the nitrogen of the ADT bridgehead were estimated through PyMOL,^31^ analyzing the following crystal structures, with pdb codes: 4XDC, 6N59, 6N6P, 6NAC, 3C8Y for *Cp*I and 6GLY, and 6GLZ for C299A and C299D, respectively. Alphafold modeling of *Tam*HydS (RMSD = 3.692 Å vs *Cp*I) revealed an overall similar structure albeit with differences in estimated distances between the proposed PTP residues (1.8 ± 0.7 Å, Figure S2).

### Site-Directed Mutagenesis

The gene encoding for *Tam*HydS was synthesized with a C-terminal Strep-Tag^II^ sequence and cloned in pET-11a(+) by Genscript using restriction sites *NdeI* and *BamHI* following codon optimization for expression in *Escherichia coli*. Parental plasmid harboring the wild-type gene was amplified by PCR with Phusion High-Fidelity to generate site-directed mutagenesis variants. Mutagenic primers were synthesized and purified by Eurofins Genomics (Ebersberg, Germany) for each of the mutants, E252V: forward 5′-GGCGTTGCGTTCGGTACCTTTAC-3′, reverse 5′-GAACGCAACGCCACCGCTG-3′; E252D: forward 5′-GGCGATGCGTTCGGTACCTTTAC-3′, reverse 5′-GAACGCATCGCCACCGCTG-3′; E289A: forward 5′-GATTTCTTTGCGGGCCTGGCGTG-3′, reverse 5′-GGCCCGCAAAGAAATCCAGGTCG-3′; E289D: forward 5′-GATTTCTTTGATGGCCTGGCGTG-3′, reverse 5′-GGCCATCAAAGAAATCCAGGTCG-3′; The PCR product was then digested with *DpnI* restriction enzyme. Gene integrity was verified via sequencing by Eurofins Genomics.

### Generation of [2Fe]^ADT^-Activated *Tam*HydS Variants

Protein purification, reconstitution of [4Fe–4S] clusters, and activation with [2Fe]^ADT^ to generate the holo-forms of the variants were performed as previously described with minor modifications.^9^ In short, the expression constructs with verified sequences were transformed in chemically competent *E. coli* BL21(DE3) or BL21(DE3)ΔiscR^32^ cells (Table S1) to express the apo-forms of *Tam*HydS lacking the di-iron subsite of the H-cluster (Figure S3). The proteins were purified via StrepTrap chromatography (StrepTrap HP (GE Healthcare) affinity column), and prior to the elution step, the column was washed with a sub-denaturing concentration of 1 M urea in 100 mM Tris, 150 mM NaCl pH 8.0.^33^ Comparing preparations of wild-type *Tam*HydS (*Tam*HydS WT) showed that the urea wash step did not change the specific activities, Fe/protein content, and spectroscopic properties as compared to preparations isolated in strict absence of urea. The yields following purification by StrepTrap chromatography varied from approximately 1–7 mg L^–1^ of cell culture depending on the variant. Subsequent operations after cell growth and induction were carried out under anaerobic conditions in the glovebox to prevent hydrogenase inactivation by atmospheric oxygen. The [4Fe–4S] clusters were reconstituted semienzymatically with cysteine desulfurase (*E. coli* IscS) (Figure S4). To generate the holo-forms of the variants for in vitro H_2_ evolution/oxidation assays, ATR-FTIR, and EPR analyses, the enzymes were mixed with [2Fe]^ADT^. After incubating the reaction mixture for 2 h, the mixture was desalted with 10 mM Tris–HCl, 2 mM sodium dithionite pH 8.0. The activated variants were then transferred into airtight serum vials before they were flash-frozen in liquid N_2_ and stored at −80 °C until further use.

### H_2_-Evolution Assays

The fully reconstituted holo-enzyme was diluted to 1 μM in 180 μL phosphate buffer (100 mM, pH 6.8) in an 800 μL crimp top vial and sealed with a natural rubber/clear PTFE septum preassembled in an aluminum cap (8 mm). The reaction was initiated with the rapid addition of 10 mM methyl viologen(MV) as the electron mediator, and 100 mM sodium dithionite as the reducing agent/sacrificial electron donor.^34^ The mixture was incubated at 25 °C and 120 rpm. After 15 min, a 100 μL aliquot from the headspace (total volume of 620 μL) was injected into a PerkinElmer Clarus 500 gas chromatograph (GC) equipped with a thermal conductivity detector and a stainless-steel column packed with Molecular Sieve (60/80 mesh). The operational temperatures of the injection port, the oven, and the detector were 100, 80, and 100 °C, respectively. Argon was used as the carrier gas at a flow rate of 35 mL min^–1^. To determine the amount of H_2_ associated with the peak area at around 0.4 min in the chromatogram, a calibration curve made up of standard points from 0 to 60% H_2_ in the 620 μL headspace was generated. One unit (U) of activity catalyzes 1 μmol of H_2_ evolved per min under the indicated assay conditions, whereas specific activity is U per mg of the enzyme.

### H_2_-Oxidation Assays

Benzyl viologen (BV) was used as the redox mediator. The absorbance of the mixture containing <30 μg of holo-enzyme and 1 mM BV in H_2_-saturated 100 mM phosphate buffer pH 6.8 was measured at 550 nm in 1 mL plastic cuvettes. A standard curve was generated to determine the molar extinction coefficient of reduced BV (ε_550_^red^ = 9.12 mM^–1^ cm^–1^). The BV specific activity (U/mg) was measured by the initial rate of change of absorbance at 550 nm where one unit (U) of activity catalyzes 2 μmol of BV reduced per min (1 μmol of H_2_ oxidized per min) under the indicated assay conditions.

### Attenuated Total Reflectance Fourier-Transform Infrared (ATR-FTIR) Spectroscopy

A solution of 1 μL enzyme (0.2–1 mM *Tam*HydS variants) in 10 mM Tris buffer (pH 8.0) was deposited on the ATR crystal in the anaerobic atmosphere of a Braun Glove box. The ATR unit (BioRadII from Harrick) was sealed with a custom build PEEK cell that allowed for gas exchange and illumination (similar to Stripp^35^ and Senger et al.^36^) mounted in an FTIR spectrometer (Vertex V70v, Bruker). The sample was dried under 100% nitrogen gas and rehydrated with a humidified aerosol (100 mM Tris–HCl, pH 8.0) as described before.^37^ Spectra were recorded with 2 cm^–1^ resolution, a scanner velocity of 80 Hz, and averaged of varying number of scans (mostly 1000 Scans). All measurements were performed at ambient conditions (room temperature and pressure, hydrated enzyme films). Gases (N_2_, H_2_, CO) were applied at a flow rate of 0.5–1.5 L/min. D_2_O exchange was performed as reported previously.^35^ Selected samples were mixed 1:1 with a 20 mM sodium dithionite (NaDT) solution, resulting in at least 10-fold excess of NaDT (Figure S5). The data were analyzed and plotted to our protocols described previously.^38^

### EPR Spectroscopy

X-band EPR measurements were performed on a Bruker ELEXYS E500 spectrometer equipped with a SuperX EPR049 microwave bridge and a cylindrical TE_011_ ER 4122SHQE cavity in connection with an Oxford Instruments continuous flow cryostat. Measuring temperatures were achieved using liquid helium flow through an ITC 503 temperature controller (Oxford Instruments). The Xepr software package (Bruker) was used for data acquisition and processing. EasySpin software version easyspin-6.0.0-dev.51 was used for spectral simulation and fitting.^39^ EPR samples of E252V and E289D were prepared in 100 mM Tris–HCl, pH 8.0 under a neat argon atmosphere and either directly flash-frozen (“as prepared”) or flushed with H_2_ or CO gas inside the EPR tube for an hour prior to freezing (“H_2_- or CO- flushed”). EPR samples incubated with D_2_ were prepared in the absence (4 μL of 1 mM enzyme in 10 mM Tris–HCl, pH 8.0 + 76 μL 100 mM Tris–HCl, pH 8.0) or presence of 95% D_2_O (4 μL of 1 mM enzyme in 10 mM Tris–HCl, pH 8.0 + 76 μL D_2_O; Figure S6). For samples that were reduced with NaDT, 1 μL of 100 mM stock solution of NaDT was added into 79 μL of 50 μM enzyme, resulting in at least 20-fold molar excess of NaDT (Figure S7).

## Results

### In Silico Analysis

To identify the amino acids that could constitute the PTP of Group D *Tam*HydS, the YASARA-generated homology model^9^ of *Tam*HydS was aligned with the crystallographic structure of Group A representative *Cp*I (pdb: 4XDC),^10^ with an RMSD of 1.541 Å (Figure S1). Amino acid residues potentially involved in proton transfer were identified by screening all residues with H-bonding capacity around a 5 Å distance, starting from the ADT of the H-cluster and progressing to the surface of the enzyme. The E252 residue (*Tam*HydS numbering unless otherwise stated) is the sole candidate for proton transfer to the H-cluster with a distance of 3.4 Å to the ADT moiety (Figure 1). As the closest protonatable residue to E252, we identified E289 (3.5 Å). From E289, the next residue identified was S249 (4.1 Å to E289). From S249, the cut-off was extended up to 5.3 Å since there were no protonatable side chains within 5 Å, resulting in D265 as the last proton transfer relay closest to the enzyme surface (5.2 Å to S249). Starting from the H-cluster, the proposed PTP of *Tam*HydS thus consists of E252 → E289 → S249 → D265. The distances between E289, S249, and D265 are slightly longer than the expected H-bonding distance. However, the model does not include intraprotein waters which can likely compensate for and modulate the elongated distances between E289, S249, and D265, as seen in structures of the PTP in Group A [FeFe] hydrogenases.^11,40^

To support the PTP proposed from the structural analysis, sequences from Group D were aligned to generate a consensus sequence, which defines the most common amino acid residues at positions 252, 289, 249, and 265 of *Tam*HydS. The alignment of multiple Group D member sequences shows that the amino acids most proximal to the H-cluster, E252 and E289, are highly conserved (Figure 2). Similarly, the final residue of the proposed pathway (D265) is also well-conserved, while the intermediate residue (S249) is moderately conserved, showing a strong preference for residues with hydroxy groups (i.e. serine and threonine). These amino acids are not conserved in Group A, B, or C, with the exception of the glutamate at position 289. Thus, our sequence alignment analysis strongly indicates that the potential PTP identified here is exclusive for Group D [FeFe] hydrogenases.

### Preparation of Variants and Their Catalytic Properties

*Tam*HydS amino acids E252 and E289 were exchanged conservatively to aspartic acid (E252D, E289D) and nonconservatively to valine or alanine (E252V, E289A) through site-directed mutagenesis to assess their role in H_2_-processing activity rates. Valine was selected for position 252 as this is found in the corresponding position of prototypical Group A hydrogenases (Figure 2), while alanine was selected for position 289 as a classical “loss-of-function” variation. The variants were expressed heterologously in *E. coli* and anaerobically purified to produce the *apo*-variants that lack the di-iron subsite (Figure S3 for gels). After reconstituting the four [4Fe–4S] clusters (Figure S4 for UV–vis spectra of the reconstituted enzyme, Table S1 for iron quantification before and after reconstitution), the *apo*-variants were mixed with the synthetic [2Fe]_H_ subsite precursor [Fe_2_(μ-ADT)(CO)_4_(CN)_2_](Et_4_N)_2_ ([2Fe]^ADT^) to generate the corresponding *holo*-variants. Although no reference is available of *Tam*HydS isolated from its native host, this is expected to yield biologically relevant samples considering the well-conserved nature of the H-cluster maturation machinery (HydEFG).^9^

**Figure 3:**
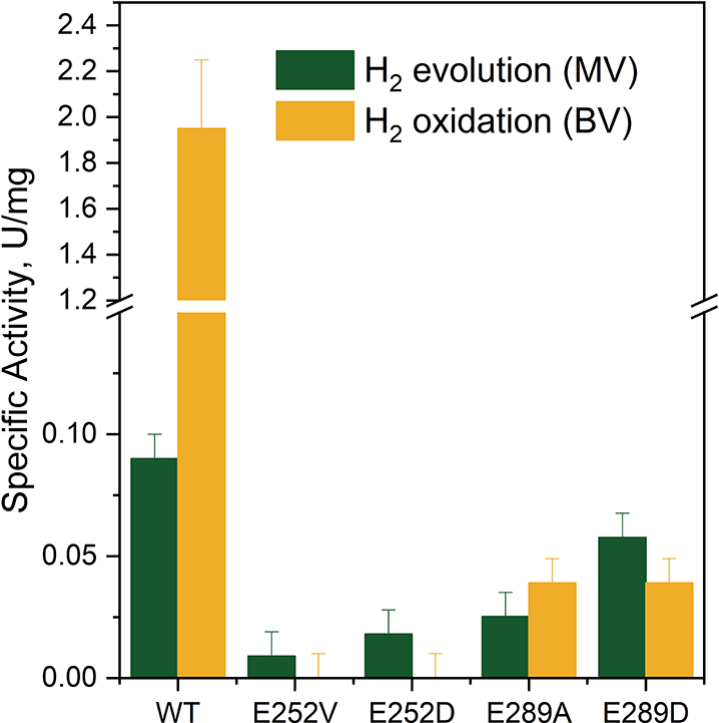
Comparison of the specific H_2_-oxidation and -evolution activities of *Tam*HydS variants quantified using benzyl viologen (BV, *E*° = −359 mV vs SHE) and methyl viologen (MV, *E*° = −446 mV vs SHE) as external redox partners versus the wild-type *Tam*HydS enzyme (WT) (note the break of the Y-axis). All reactions were performed in 100 mM potassium phosphate buffer pH 6.8. H_2_ oxidation (yellow bars) was measured by reducing 1 mM BV at 550 nm in H_2_-saturated buffer. The amount of H_2_ produced from 10 mM MV + 100 mM sodium dithionite was measured by gas chromatography (green bars).^34^ Specific activity (U/mg) is one unit (U) of activity that catalyze 1 μmol of H_2_ oxidized/evolved per min under the indicated assay conditions over the amount of enzyme in the reaction mixture (mg). The absolute values of the specific activities are tabulated in Table S2. Error bars indicate standard error, with *n* = 6 (three technical repeats each for two biological samples).

The effects of *Tam*HydS variants E252V, E252D, E289A, and E289D on the rates for reduction of H^+^ (H_2_ evolution) and oxidation of H_2_ were studied in solution assays, each performed in pH 6.8 and at 25 °C ( = −403 mV vs SHE). Methyl viologen (MV, *E*° = −446 mV vs SHE) and benzyl viologen (BV, *E*° = −359 mV vs SHE) were employed as the electron donor and acceptor, respectively. All the variants negatively impacted the H^+^/H_2_-interconversion activities compared to the wild-type *Tam*HydS enzyme (*Tam*HydS WT) (Figure 3). H_2_-oxidation was the most severely affected, with E252V/D and E289A/D displaying <0.01 and ≤3% residual activities, respectively. In terms of H_2_-evolution, the nonconservative (E252V) and conservative (E252D) substitutions are the least functional variants with specific activities that range from 10 to 20% with respect to *Tam*HydS WT. On the other hand, the conservative exchange of E289 to glutamate had the least effect (E289D retaining 70% residual activity). This is followed by the nonconservative exchange to an alanine (E289A) at 30% residual activity. In general, the substitution of E252 had the greater impact in both catalytic directions, most likely since it is closer to the ADT ligand than E289. A similar decrease in activity has been reported for variants of Group A hydrogenases where amino acids in their PTP were varied.^11,41^

In addition to the change in overall activity, it is also noteworthy that the variants display a distinct change in apparent catalytic bias. Under the employed solution assay conditions, the *Tam*HydS WT displays significantly higher rates for H_2_ oxidation than H^+^ reduction. However, this bias is suppressed in all studied variants and even switched to an apparent bias toward H^+^ reduction in three cases (variants E252V/D and E289D).

### Spectroscopic Properties

#### FTIR Spectroscopy

**Figure 4:**
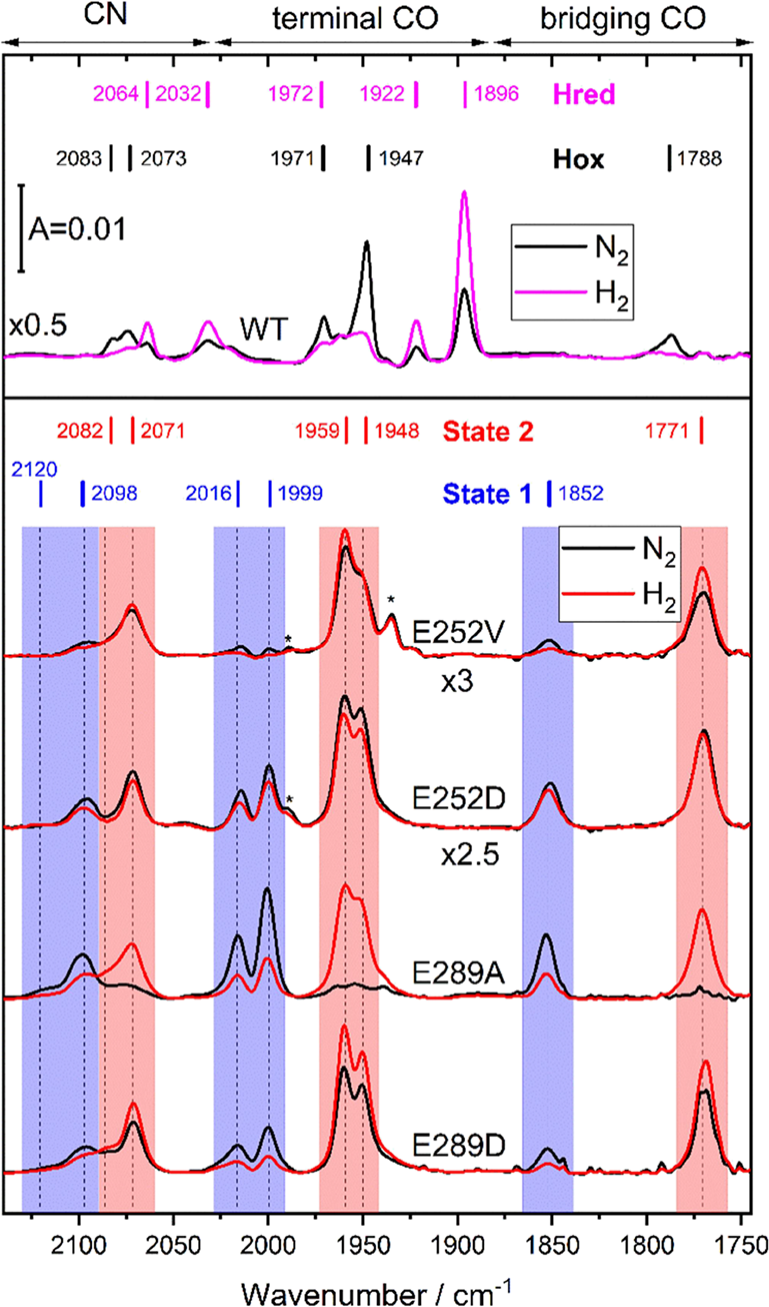
FTIR spectra of wild-type *Tam*HydS and variants exposed to N_2_ and H_2_. For *Tam*HydS wild-type (WT) and each variant, the FTIR spectra under N_2_ (black spectra) and H_2_ atmosphere (magenta and red spectra, respectively) are shown. *Tam*HydS WT accumulates the oxidized state (H_ox_) under a nitrogen atmosphere (top black spectra) and the reduced state (H_red_) under a reducing hydrogen atmosphere (top magenta spectrum) (data shown taken from ref (9).). In the *Tam*HydS variants, two IR signatures indicative of different redox states are observed. **State 1** with bands at 2120, 2098, 2016, 1999, and 1852 cm^–1^ (indicated with blue vertical bars) and **State 2** shifted to lower energies (red-shifted) with bands at 2082, 2071, 1959, 1948, and 1771 cm^–1^ (indicated with red vertical bars). Upon H_2_ exposure (red spectra), **State 2** is populated in varying fractions. The E252 variants show only diminutive changes (see also Figure S9). The redox state distribution in the E289 variants clearly shifts to **State 2** upon H_2_ exposure. Peaks of unassigned H-cluster species are labeled with an *.

The effect of each amino acid variation on the H-cluster was examined using ATR-FTIR spectroscopy. Enzyme solutions were dried on the ATR crystal surface and rehydrated under a humidified N_2_ aerosol as reported previously.^37^*Tam*HydS WT accumulates the oxidized state (H_ox_) under a nitrogen atmosphere (Figure 4 top black spectrum) and the reduced state (H_red_) under a reducing hydrogen atmosphere (Figure 4 top magenta spectrum).^9^ No distinct features attributable to either of these two states were observed in any of the variants studied here. Instead, the variants showed two FTIR signatures indicative of redox states previously not identified in prototypical [FeFe] hydrogenase (Figure 4 bottom spectra and Figure S8). The states are denoted as **State 1** with bands at 2120, 2098, 2016, 1999, and 1852 cm^–1^ (blue bars Figure 4), and **State 2** shifted to lower energies (red-shifted) with bands at 2082, 2071, 1959, 1948, and 1771 cm^–1^ (red bars Figure 4). We note additional small peaks (indicated by *) from minor H-cluster species that we were unable to assign completely. Under an N_2_ atmosphere, the nonconservative exchanges of E252 and E289 appeared to stabilize specific states. E252V was found to predominantly yield **State 2**, while E289A was found to be predominantly in **State 1** (Figure 4 bottom black spectra). Meanwhile, both the conservative exchanges of E252D and E289D showed a mixture of **State 1** and **State 2**, with a slight preference for the latter state.

Exposure to a H_2_ (1 atm) atmosphere led to a clear shift in the relative redox state population in favor of **State 2** in the E289 variants. The E252 variants displayed only a minor (E252V) or no (E252D) shift of the redox state equilibrium toward **State 2** (Figure 4 bottom red spectra). The stronger response to H_2_ exposure of the E289 variants correlates with their observed H_2_ oxidation capability, compared to the undetectable H_2_ oxidation activities of the E252 variants (Figure 3). Notably, while *Tam*HydS WT has a low CO affinity,^9^ none of the variants bind external CO to form the inhibited “H_ox_-CO” state (data not shown). Instead, the exposure to CO led to a slow conversion of the enzyme population from **State 2** to **State 1** (representative spectra for E252V are shown in Figure S9C,D). Reduction using sodium dithionite caused a shift toward **State 2** (Figure S5).

The observed red-shift of cofactor ligand bands upon H_2_ exposure (approximately Δ30 cm^–1^ for CN and Δ50–80 cm^–1^ for CO ligands) indicates a reduction at the di-iron site when compared to similar shifts observed for Group A [FeFe] hydrogenases.^42^ For *Tam*HydS WT, the CN band intensities for H_ox_, H_red_ (Figure 4 top spectra) and H_ox_-CO states are more or less even.^9^ In contrast, for both **State 1** and **State 2**, the area of the lower wavenumber CN band (2098 and 2071 cm^–1^ for **State 1** and **State 2**, respectively) exceeds the area of the higher wavenumber CN bands (2120 and 2082 cm^–1^) greatly, suggestive of a difference in the hydrogen bonding strength of the CN ligands and/or an alteration in cofactor geometry or ligand binding.^43^ Moreover, for **State 2** the relative band area of the two terminal CO ligands (1959 and 1948 cm^–1^) are inverted (larger band area for the higher wavenumber band) when compared to all redox states known for *Tam*HydS WT and **State 1** (2016 and 1999 cm^–1^) hinting in a similar direction (Figure 4). Another peculiarity of the band pattern of **State 2** is the large distance between the terminal CO ligand bands (1959, 1948 cm^–1^) and the band assigned to μCO (1771 cm^–1^). Although **State 2** accumulates under reducing conditions, H/D exchange revealed no specific shifts of cofactor ligand bands for **State 1** or **State 2** indicative of e.g., (terminal) hydride binding (Figure S5).^41,44^

#### EPR Spectroscopy

**Figure 5:**
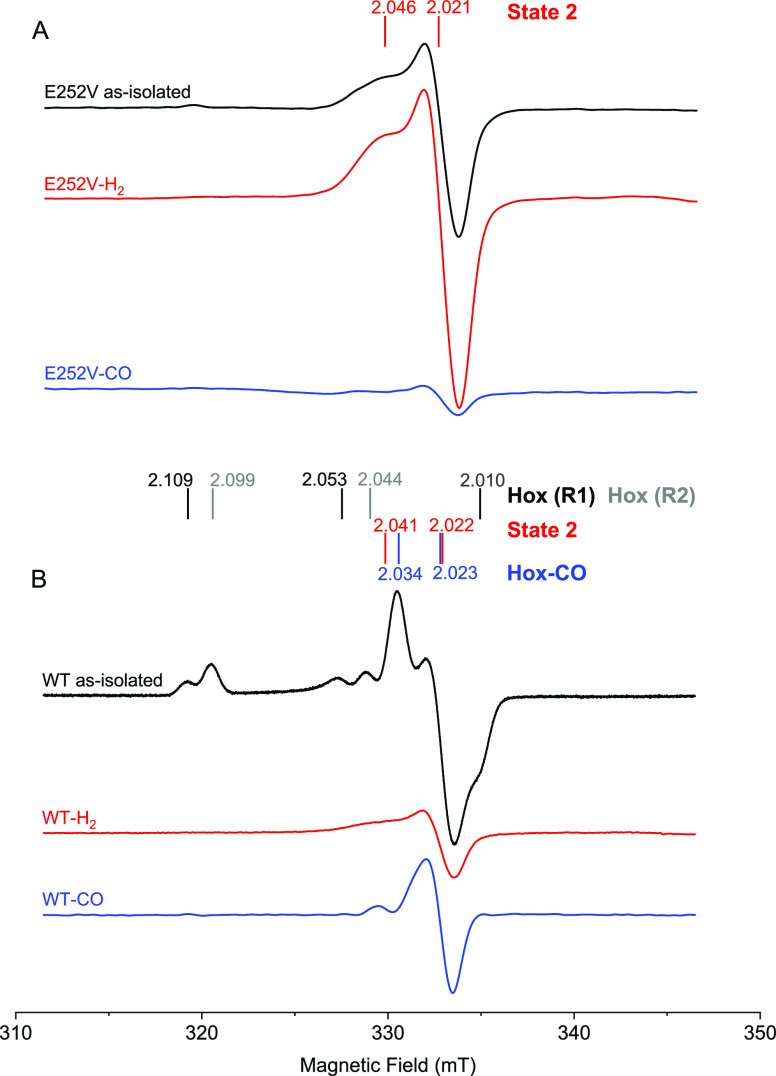
EPR spectra of *Tam*HydS variant E252V and wild-type (WT). (A) EPR measurements of E252V prepared under a neat argon atmosphere (“as-isolated,” black spectrum) revealed a dominant pseudo-axial signal, assigned to **State 2** (*g*_∥_ = 2.046 and *g*_⊥_ = 2.021), which increases after H_2_-flushing (red spectrum) and disappears after flushing with CO (blue spectrum). (B) As-isolated spectrum of *Tam*HydS WT (black) reveals rhombic features of the H-cluster in the H_ox_ states (two components denoted as R1 and R2 with the corresponding *g*-values in black and gray respectively) and an axial feature corresponding to H_ox_-CO (*g*-values in blue). *Tam*HydS WT samples incubated under H_2_ (red spectrum) and CO (blue spectrum) shown for reference. Data adapted from Land et al.,^9^ revising the *g*-values assigned to H_ox_-CO (*g*_∥_ = 2.034 and *g*_⊥_ = 2.023) and assigning the previously unknown signal that appears when WT is flushed with H_2_ to **State 2** (*g*_∥_ = 2.041 and *g*_⊥_ = 2.022). Spectra recorded with the following settings: *T* = 21 K; modulation frequency = 100 kHz; amplitude = 10 G; microwave frequency = 9.4 GHz; microwave power = 16 μW.

The electronic configuration of **State 1** and **State 2** was further probed using EPR spectroscopy. The E252V variant was selected as a representative example as it displayed a high fraction of **State 2**. X-band EPR spectra recorded at 21 K of this “as-isolated” E252V sample were dominated by a narrow axial signal, (Figure 5A, black spectrum). This is in contrast to reference EPR spectra recorded of analogously prepared samples of *Tam*HydS WT (Figure 5B), which shows a mixture of oxidized resting states associated with two H_ox_ components and a CO-inhibited state H_ox_-CO in the *g* ∼ 2 region (Table S3).^9^

The amplitude of the axial signal in E252V was further increased after continuous flushing with H_2_ (Figure 5A, red spectrum). Consequently, it is assigned to **State 2**, in accordance with the red-shifted features observed by FTIR spectroscopy following H_2_ treatment. Simulation of the **State 2** EPR signal showed that it can be described as a pseudo-axial EPR signal with *g*_∥_ = 2.046, *g*_⊥_ = 2.0225, 2.0196 (average 2.021) (Figure S10). In addition to the narrow axial signal, spectra of H_2_-treated samples of E252V collected at 10 K also displayed features attributable to reduced F-clusters ([4Fe–4S]^+^) (Figures S10 and S11). This shows that the H-cluster remains capable of shuttling electrons into the accessory clusters despite modifications to the putative PTP. As observed by ATR-FTIR spectroscopy, observation of **State 2** is not strictly dependent on H_2_ treatment, as the same signal was found in E252V (and E289D) when treated with sodium dithionite (Figure S5 and S7). Finally, apart from variations in relative intensities, no distinct differences in the signal shape were observed for the **State 2** EPR signal in E252V and E289D when comparing samples generated under H_2_ to D_2_, in the absence or presence of 95% D_2_O in the buffer (Figure S6).

Continuous flushing of E252V samples under a neat CO atmosphere resulted in a significant decrease in the amplitude of the axial EPR signal, with no new distinct features appearing in the spectrum (Figure 5A, blue spectrum). Notably, E252V did not show any signs of CO-inhibited state, H_ox_-CO, even when it was flushed with CO gas for an hour. This supports the notion that CO treatment induces the formation of **State 1** (Figure 5A, blue spectrum) as seen by FTIR spectroscopy (Figure S9C,D) and that **State 1** is EPR-silent.

In summary, the data from FTIR and low-temperature EPR measurements revealed that the variants do not accumulate any states commonly observed for the H-cluster in [FeFe] hydrogenases. However, we note that an axial signal strikingly similar to that of **State 2** was previously reported for *Tam*HydS WT (Figure 5 and Table S3).^9^

## Discussion

Based on studies of Group A enzymes, [FeFe] hydrogenases are generally considered to be fast and reversible catalysts, highly sensitive to CO inhibition.^1a,2a,12a^ The recent characterization of Group C and D [FeFe] hydrogenases has shown that this is not always the case.^9,16b,19^ Elucidating key reactivity differences between [FeFe] hydrogenases and the structural factors promoting this is arguably central to our understanding of biological H_2_-metabolism. In parallel, it is expected to provide guiding principles in the preparation of molecular-based catalysts for redox catalysis.

Through homology modeling and sequence alignment, we have now identified a putative PTP unique for Group D [FeFe] hydrogenases. Employing *Tam*HydS as a Group D representative, variants were generated through site-directed mutagenesis that target E252 and E289, the two putative proton relay sites most proximal to the H-cluster. The variants were characterized biochemically and spectroscopically, and all variants were found to have a significant impact on H_2_ catalysis and the H-cluster redox state configuration. In all cases, the overall rates for H_2_ oxidation and production decreased, with variation of the proposed proton relay partner of the ADT-amine (E252), having the most prominent effect. In addition, the variants also resulted in a shift in apparent catalytic bias of the enzyme toward H_2_ evolution. With regard to the latter point, arguably the most striking observation was made for E289D which retained around 70% of the H_2_ production capacity of *Tam*HydS WT, while significantly decreasing H_2_ oxidation rates (≤3% relative to *Tam*HydS WT).

In addition to decreasing catalytic rates, altering the putative PTP clearly had a direct effect on the accumulation of H-cluster states of *Tam*HydS. The variants form almost exclusively two previously unidentified states (**States 1** and **2**, Figures 4 and 5). Conversely, the redox states observed for *Tam*HydS WT and most Group A [FeFe] hydrogenases, i.e., the well-known H_ox_ and H_red_ states, are absent.^9,45^ The exact nature of the H-cluster species denoted as **State 1** and **State 2** herein remains to be fully elucidated. Still, the spectroscopy data reported here do provide initial insight. In short, **State 2** (*S* = 1/2) is an EPR-active species that forms under reducing conditions while **State 1** (*S* = 0) is EPR-silent and is relatively more oxidized. The IR band pattern of **State 1** shows some similarities with the so-called H_inact_ and H_hyd_ states previously observed in Group A [FeFe] hydrogenases (Figure S12 for the structures of the H-cluster states).^2a,41,42,46^ The EPR-silent nature of this state implies an oxidized [4Fe–4S]_H_ cluster ([4Fe–4S]_H_^2+^), yielding an overall oxidation state analogous to the previously reported inhibited “over-oxidized” H_inact_ state (i.e., [4Fe–4S]^2+^-[Fe^II^Fe^II^]),^2a,42a,46a−46e^ although we stress that the ligation of the [2Fe]_H_ subsite is not necessarily identical. The red-shift observed by FTIR spectroscopy indicates that **State 2** reflects a reduced state relative to **State 1**. Given the axial and relatively narrow nature of the H_2_-induced EPR signal, the unpaired electron likely resides on the [2Fe]_H_ subsite rather than the iron–sulfur cluster, as the latter usually displays broader rhombic signals in its reduced ([4Fe–4S]_H_^+^) state.^9,12a^ Based on these observations, we tentatively assign **State 2** a formal oxidation state analogous to that of H_ox_ (i.e. [4Fe–4S]^2+^-[Fe^I^Fe^II^]), which can explain the strong red-shift of the μCO ligand by 81 cm^–1^ in particular. However, the well-known active-ready resting-state H_ox_ generally gives rise to a rhombic EPR signal, as also reported previously in the case of *Tam*HydS WT.^9^ A somewhat related observation of unusual EPR properties of H_ox_-related states has recently been reported for the Group A [FeFe] hydrogenase from *C. beijerinkii* (*Cb*HydA1). In the latter case, two distinct EPR signals were observed for the H_ox_ state, but only a single set of FTIR bands, an observation attributed to structural flexibility around the [4Fe–4S] component of the H-cluster.^47^ Assuming that **State 2** does indeed correspond to a similar mixed valence I,II formal oxidation state of the [2Fe]_H_ subsite, the shift from rhombic to axial EPR signal as well as the distinct differences observed in the FTIR spectra indicates a change in the geometry and/or electron configuration of the H-cluster as compared to the standard H_ox_ state.^9^ Moreover, **State 2** accumulates under H_2_ in at least three of the variants (E289A/D and E252V), while incubation under a CO atmosphere results in (slow) conversion to **State 1**. This behavior is in stark contrast to what is commonly observed for the H_ox_ state, which generally converts to H_red_ and H_hyd_ species under H_2_ and H_ox_-CO under CO.

**Figure 6:**
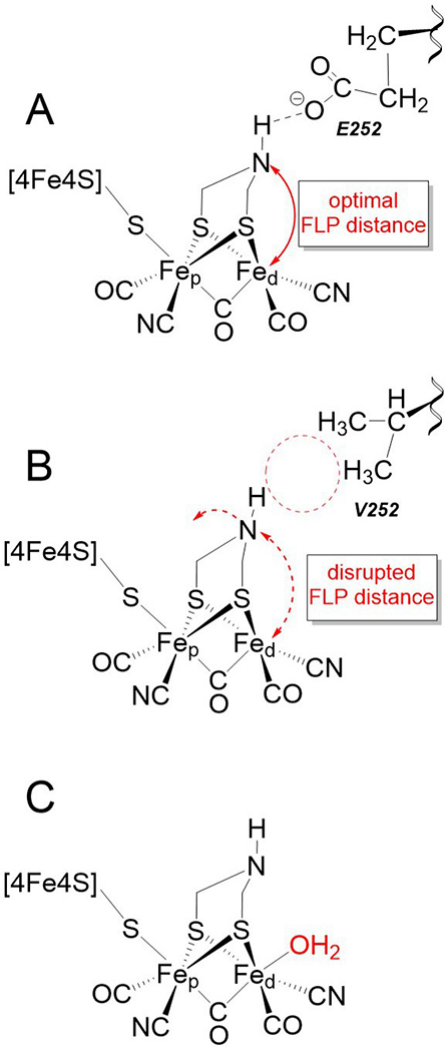
Proposed models illustrating the structural changes at the H-cluster by the PTP variants. (A) Schematic model illustrating an optimal FLP interaction between the ADT-amine and the distal Fe (Fe_d_) of the H-cluster. The glutamate at position 252 (E252) of *Tam*HydS WT likely fine-tunes the positioning of the ADT-amine, either through direct H-bonding or indirectly via intermediate water molecules, thus ensuring an optimal FLP distance from Fe_d_ for heterolytic H_2_ cleavage. (B) Absence of H-bonding provided by the carboxylate side chain upon exchange of E252 to V252 shifts the ADT-amine toward a relatively farther distance from Fe_d_, releasing cofactor strain and consequently elongates the FLP distance. (C) Reduced steric hindrance, due to a relaxed FLP geometry as illustrated in (B), could facilitate the binding of exogenous ligands to Fe_d_, thereby, facilitating the formation of inhibited states. The binding of an aqua (or hydroxide) ligand is proposed for **State 1**.

The lower catalytic rates as well as the altered properties of the H-cluster of the variants support a critical influence of the varied residues. The larger decrease of H_2_ oxidation rates is in line with an impaired proton transport affecting the faster reaction more strongly than the intrinsically slower proton reduction reaction (Figure 3). However, the effects on bias as well as stabilization of new H-cluster states are difficult to reconcile with an exclusive substrate (proton) transfer influence.^11,48^ It is well established that bias in hydrogenases can further be controlled by, e.g., accessory FeS clusters, which govern the intramolecular electron transfer rates.^22,49^ In parallel, a variation in bias has previously also been observed in Group A [FeFe] hydrogenase variants in which the terminal PTP residue (C299_*Cp*I_) was exchanged into serine or aspartate,^11,50^ an effect attributed to changes in relative acidity of the proton donor and the [2Fe]_H_ subsite.^50^ Still, also these two latter models struggle to fully rationalize the observed reactivity of the variants. Instead, we propose that the variations of the PTP studied here cause a structural change at the H-cluster, resulting in a disruption of the ADT-amine and Fe_d_ FLP interaction (Figure 6A,B). More specifically, the E252 residue is likely contributing to the stabilization of an ADT geometry where the amine is positioned at an optimal distance from Fe_d_ for heterolytic H_2_ cleavage. Removing this carboxylate (E252V), or even shortening the residue by one methylene group (E252D), releases strain from the cofactor facilitating formation of a conformation with decreased activity. Similarly, variations of the second target residue, E289, are likely to not only impair proton transfer but also influence the positioning of the ADT ligand either directly or via interactions with E252. Changes in the H-bonding network around the [2Fe]_H_ subsite could also rationalize the changes in relative intensities of the CN bands between *Tam*HydS WT and the variants, observed by FTIR spectroscopy. Hints of similar structural effects are found in crystallographic data on *Cp*I, which show that variations of the closest PTP residue to the ADT ligand (C299A_*Cp*I_ and C299D_*Cp*I_) slightly reduced the distance between the Fe_d_ and ADT-amine by 0.3–0.4 Å. Moreover, variation of the next proton transfer relay (E279D_*Cp*I_) showed modified configurations of the other PTP side chains.^11^

Beyond the geometry argument outlined above, additional contributions from changes in electrostatics cannot be excluded. However, as the site of variation (E252 or E289) had a greater impact on reactivities than the nature of variation (conservative or nonconservative), we consider electrostatics unlikely to be a dominant factor. Assuming an impaired FLP geometry in the variants, it follows that the change in bias can be motivated with different rate-determining steps depending on the direction of the catalytic cycle. During H_2_ oxidation, the heterolytic cleavage of H_2_ appears to be an important contributor to the overall rate. Conversely, the H–H bond formation step appears to contribute less during the reverse reaction, as the variants have a relatively smaller effect on the rates of H_2_ production.

As **State 1** and **2** are found to accumulate in variants with impaired function, care needs to be taken when attributing them catalytic relevance. Still, in particular the E289 variants do display significant H^+^ reduction activities despite only displaying these two states in FTIR spectroscopy almost exclusively. Moreover, our earlier study on *Tam*HydS has shown that upon treatment with H_2,_ an axial EPR signal with *g*_∥_ = 2.041 and *g*_⊥_ = 2.022 dominates the spectrum (Figure 5B).^9^ The identity of the H_2_-induced species in *Tam*HydS WT (denoted A1) reported in the study by Land et al.^9^ is most likely analogous to **State 2**. In Group A [FeFe] hydrogenases, variations in the PTP close to the H-cluster (e.g., C299_*Cp*I_) facilitate the accumulation of a terminal hydride species under a H_2_ atmosphere.^41,44^ Subsequent studies have shown that the latter state, denoted H_hyd_, accumulates also in wild-type Group A enzymes, strongly supporting its catalytic relevance.^37,41,51^ The absence of any distinct isotope effect on the FTIR and EPR spectra in the presence of D_2_ or D_2_O argues against ligation of a terminal hydride ligand in either **State 1** or **2**. Still, it is tempting to consider **State 2** as reflecting a catalytically active H-cluster state. Conversely, the H_inact_-like **State 1** is most likely an inhibited species. Assuming that **State 1** formation is associated with the binding of an extra ligand, this process would be facilitated by the removal of steric hindrance. Considering the disrupted FLP model, an increased ADT-amine and Fe_d_ distance could then also rationalize the apparent facile formation of this state. Again comparing to earlier studies of Group A [FeFe] hydrogenases, it has been reported that exchange of the terminal PTP cysteine residue to alanine facilitates the binding of exogenous cyanide ligands to the [2Fe]_H_ subsite.^52^ This was attributed to a decreased electron density at the [2Fe]_H_ subsite following the cysteine to alanine exchange, but we note that a steric rationale might also be applicable. Ligand binding to the [2Fe]_H_ subsite during **State 1** formation would also provide an explanation for its sluggish reactivity toward H_2_, as particularly noted for the E252 variants (Figure 4). As no potential inhibitor is added to the enzyme, we propose that the most likely incoming ligand would be H_2_O. **State 1** would then represent an inhibited state, coordinating an aqua or hydroxido ligand analogous to earlier models of the so-called H_inact_ state (Figure 6C).^42a,53^ Further studies are clearly needed to fully elucidate the electronic properties and ligand geometry of these states as well as their catalytic relevance.

In conclusion, this study has provided a first insight into the PTP in Group D [FeFe] hydrogenase. This study also showed how a relatively conservative change in ≥5 Å distance from the H-cluster (E289D) can significantly alter the apparent catalytic bias of the enzyme. However, the PTP effects are likely convoluted with effects on the H-cluster itself. This study also supports the notion that the route of proton transfer is not universal across phylogenetic groups of [FeFe] hydrogenases. Indeed, aligning the amino acids that are part of the proposed PTP in Group D *Tam*HydS with the corresponding amino acids in Group A, B and C reveal that they are unlikely to share a similar PTP. Why the two putatively sensory groups (Group C and D) of [FeFe] hydrogenases have major differences in their PTP remains to be resolved. When analyzing the sequences of Group C from Greening et al.^30^ and further extracting the Group D sequences from that data using their differences as presented in Calusinska et al.,^16a^ one can see that both groups are present in around 25% of all organisms (including *Thermoanaerobacter mathranii*) in that dataset. This indicates that they actually have different functions and if they are both sensory, they could sense H_2_ at different concentrations and/or induce different cellular responses. Furthermore, in some rare instances, organisms also encode multiple isozymes of Group C or Group D, which strengthens the hypothesis that one organism can encode and benefit from several sensory [FeFe] hydrogenases.

Future work aims to closely examine the role of the next amino acids proposed to complete the PTP (S249 and D265), confirm the changes in the local structure (including E252 and E289), and identify geometrical considerations of the hydrogen bonding network through high-resolution structural studies. The identification of this possible alternative PTP paves the way to elucidate how the protein environment promotes the diverging reactivity of different groups of [FeFe] hydrogenase. By extension, this can be expected to expand the tool-kit in the design of site-isolated catalysts coupling proton transfer and redox chemistry.

## Supporting Information Available

Alignment of *Cp*I and *Tam*HydS, SDS-PAGE gels, details on FeS cluster reconstitution and Fe content of *Tam*HydS variants, tabulated specific activity data, as well as additional FTIR and EPR spectroscopy data, and schematic representations of proposed catalytic cycles of [FeFe] hydrogenase (PDF)
