# Supplementary material for: Probing Substrate Transport Effects on Enzymatic Hydrogen Catalysis: An Alternative Proton Transfer Pathway in Putatively Sensory [FeFe] Hydrogenase
Source: ACS Catal. 2023 Jul 26;13(15):10435–46. doi: 10.1021/acscatal.3c02314 (PMC10407848; doi:10.1021/acscatal.3c02314)
Supplement: Supplementary file 1 — cs3c02314_si_001.pdf [file cs3c02314_si_001.pdf]

Supplementary Information

Probing substrate transport effects in enzymatic hydrogen catalysis - An alternative proton transfer pathway in putatively sensory [FeFe] hydrogenase

Princess R. Cabotaje[a †], Kaija Walter[a †], Afridi Zamader[a], Ping Huang[a], Felix Ho[a], Henrik Land[a], Moritz Senger[a,*], Gustav Berggren[a, *]

[a] Molecular Biomimetics, Department of Chemistry, Ångström Laboratory, Uppsala University, Box 523, SE-75120, Uppsala, Sweden

[†] P.R.C. and K.W. contributed equally to this work

*Email: gustav.berggren@kemi.uu.se
moritz.senger@kemi.uu.se

Table of Contents

Figure S1. Alignment of *Cp*I and *Tam*HydS 3

Figure S2. Comparison of *Tam*HydS Alphafold and homology models 4

Figure S3. SDS-PAGE Analysis of apo-*Tam*HydS variants 5

Figure S4. Reconstitution of [4Fe-4S] clusters 6

Table S1. Irons per protein content before and after reconstitution 7

Table S2. Specific activities by the holo-*Tam*HydS variants 8

Figure S5. FTIR spectra of *Tam*HydS variants prepared with excess sodium dithionite and H/D exchange for State 1 and State 2 9

Figure S6. EPR spectra of holo-*Tam*HydS E252V and E289D variants after treatment with D_2, with and without D_2O. 10

Figure S7. EPR spectra of holo-*Tam*HydS E252V and E289D variants with and without sodium dithionite (NaDT) treatment. 11

Figure S8. FTIR difference spectra of E289A exposed to N_2 and H_2 12

Figure S9. FTIR difference spectra and redox state population kinetics of *Tam*HydS variant E252V exposed to H_2 and CO 13

Figure S10. Experimental versus simulated EPR spectra of **State 2** 14

Figure S11. EPR spectra visualizing the $[4Fe\text{-}4S]^+$ clusters present in H_2-reduced samples of the holo-*Tam*HydS E252V variant. 15

Table S3. Tabulated g-values of the H-cluster of holo-*Tam*HydS WT and E252V and E289D variants. 16

Figure S12. Models for the catalytic cycle of [FeFe] hydrogenases 17

References: 18

Figure S1. Alignment of *Cp*I and *Tam*HydS

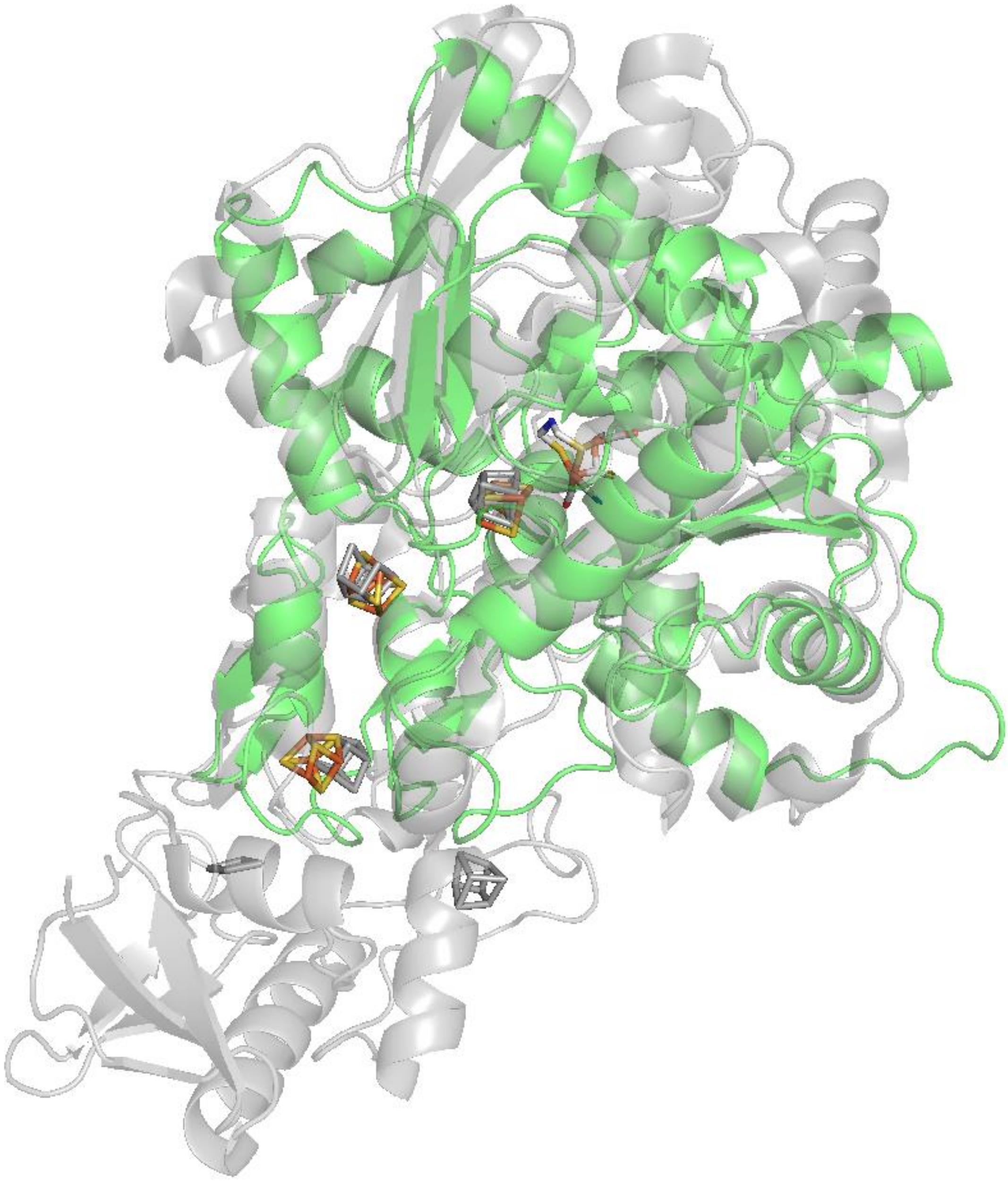

Figure S1. Alignment of the X-ray crystal structure of *Cp*I (pdb: 4XDC)[1] **and YASARA-generated homology model of *Tam*HydS[2].** The structures are represented as cartoon in gray and green for *Cp*I and *Tam*HydS, respectively. The H-cluster is represented as sticks with the following coloring scheme: Carbon, gray; nitrogen, blue; oxygen, red; sulfur, yellow; iron, orange. The iron sulfur clusters are represented as gray and yellow + orange for *Cp*I and *Tam*HydS, respectively. The alignment resulted in an RMSD of 1.541 Å. Alphafold modelling of *Tam*HydS (RMSD = 3.692 Å vs *Cp*I) revealed an overall similar structure albeit with differences in estimated distances between the proposed proton transfer pathway residues (1.8 ± 0.7 Å), see also Figure S2.

Figure S2. Comparison of *Tam*HydS Alphafold and homology models

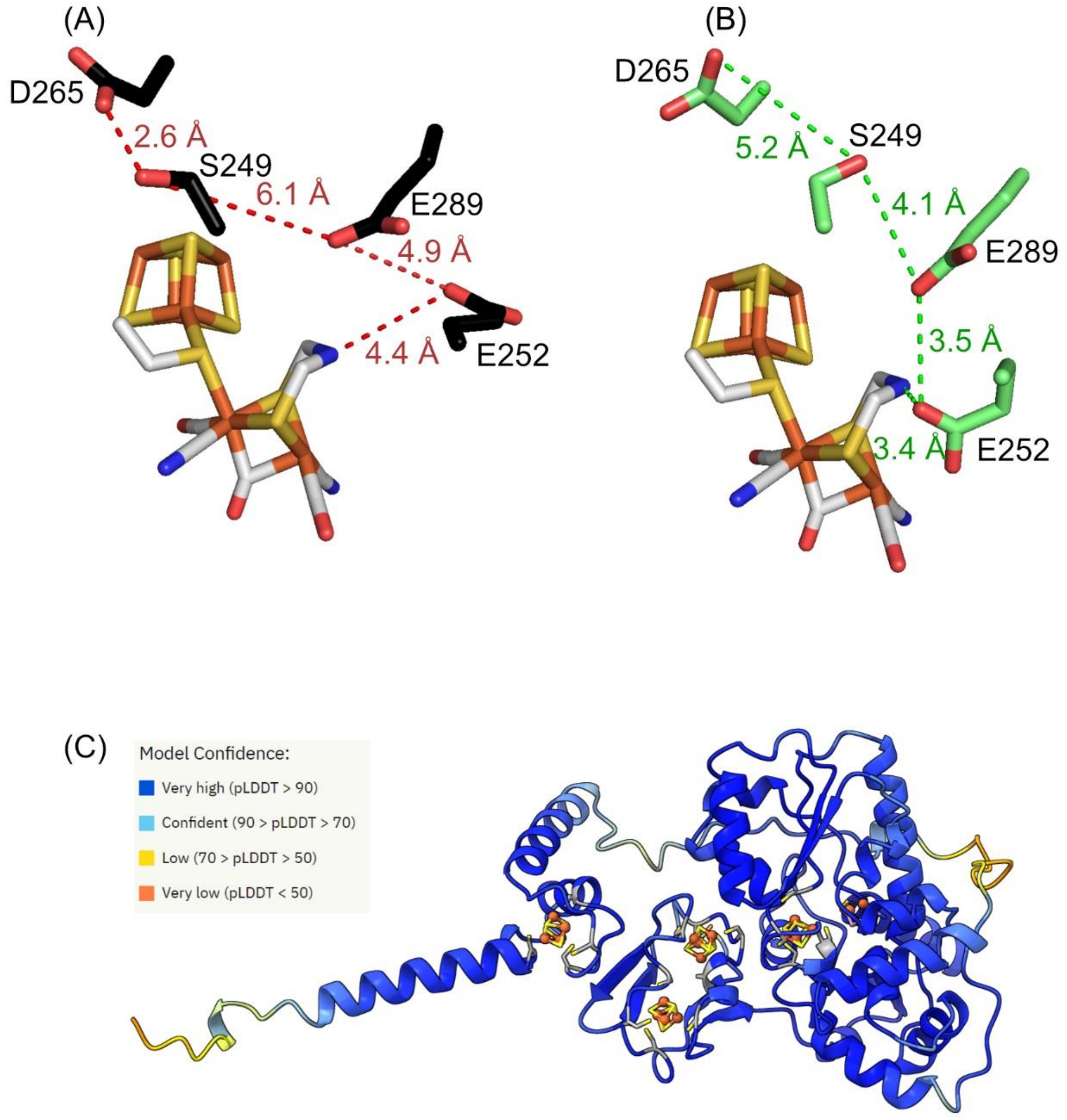

Figure S2. Comparision of *Tam*HydS Alphafold and homology models. (A) Positions of the proposed proton transfer pathway residues from the AlphaFold model of *Tam*HydS (RMSD = 3.692 Å vs *Cp*I, pdb: 4XDC)[1] (B) Positions of the proposed proton transfer pathway residues from the YASARA-generated model of *Tam*HydS (RMSD = 1.541 Å vs *Cp*I) (C) Predicted lDDT per position, showing the model confidence (out of 100) at each position of the Alphafold model shown in (A). The difference of distances between Alphafold and homology model is on average: 1.8 ± 0.7 Å. The difference of distance per PTP residue: E252 1.0 Å; E289 1.4 Å; S249 2.0 Å; D265 2.6 Å.

Figure S3. SDS-PAGE analysis of apo-*Tam*HydS variants

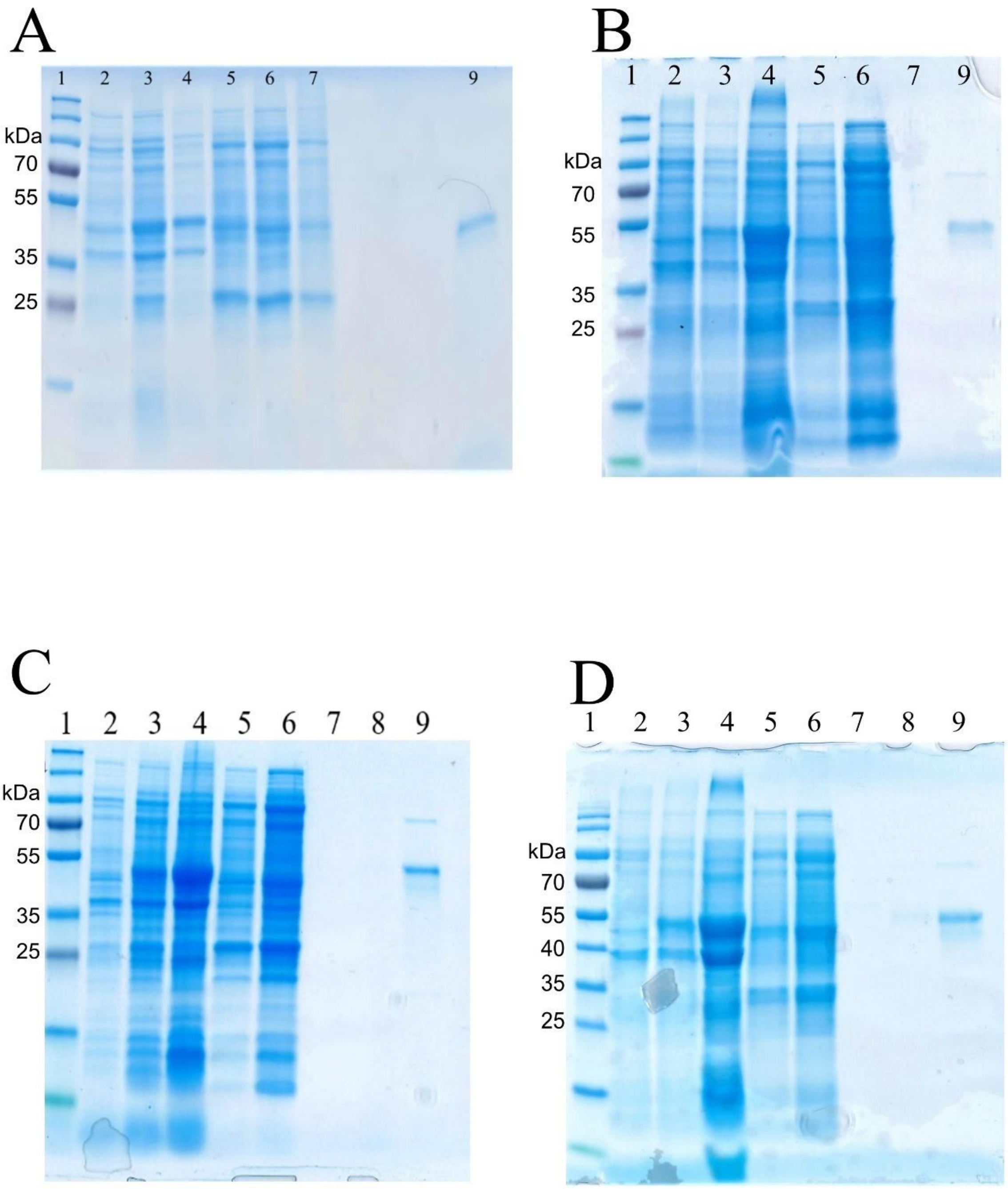

Figure S3. SDS-PAGE gels of expressed and purified apo-*Tam*HydS variants. (A) E252V (B) E252D (C) E289A (D) E289D. Lane 1: protein standard ladder (For A-C: Thermo Scientific™ PageRuler™ Plus Prestained Protein Ladder 10 to 250 kDa, For D: Thermo Scientific™ PageRuler™ Prestained Protein Ladder 10 to 180 kDa); Lane 2: *E.coli* cells before induction; Lane 3: after induction; Lane: 4 lysis pellet; Lane 5: lysis supernatant; Lane 6: Strep-trap column-flow through; Lane 7: binding buffer; Lane 8: urea wash; Lane 9: Strep-trap elution buffer.

Figure S4. Reconstitution of [4Fe-4S] clusters

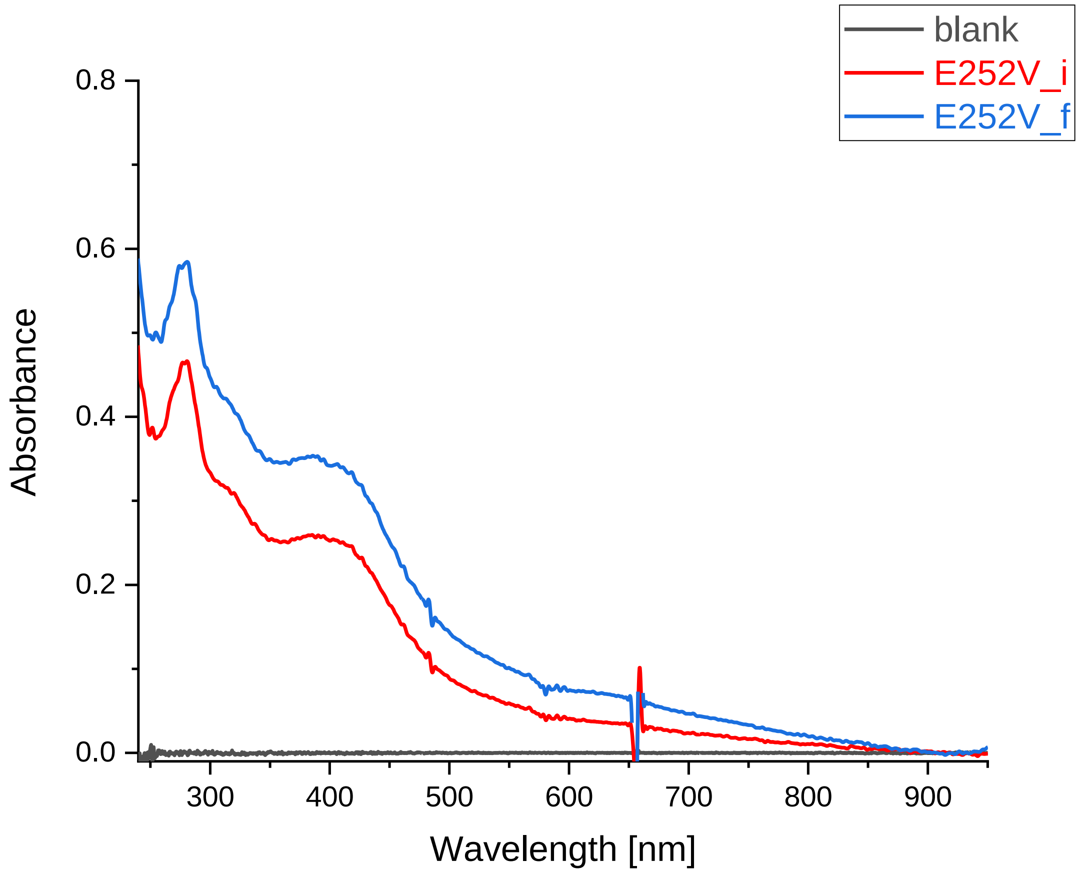

Figure S4. Representative spectra on the reconstitution of [4Fe-4S] clusters of apo-*Tam*HydS variant E252V. An increase of absorbance at 405 nm indicates formation of $[4Fe\text{-}4S]^{2+}$ clusters. The as purified apo-variant was incubated with dithiothreitol (DTT) anaerobically for 10 min at room temperature prior to the addition of ferrous ammonium sulfate and L-cysteine, and recombinant cysteine desulfurase (*E. coli* IscS). Shown is the representative UV/Vis spectra of 50 µM E252V in 100 mM Tris-HCl, 150 mM NaCl pH 8.0 before (E252V_i, red) and after reconstitution (E252V_f, blue), measured in a 1 mm cuvette.

Table S1. Irons per protein content before and after reconstitution

Variant	Fe/Protein	
	Before Reconstitution	After Reconstitution
**E252V***	10.3 ± 0.9	16.0 ± 0.2
**E252D***	8.0 ± 0.1	16.0 ± 0.1
**E289A****	16.1 ± 0.1	N/A
**E289D****	16.0 ± 0.1	N/A
**WT[2]***	13.9 ± 0.9	16.1 ± 0.3

Table S1. Fe:protein content before and after reconstitution. The presence of 16 Fe/protein indicates the expected four [4Fe-4S] clusters. The asterisk (*) indicates isolation following expression in BL21(DE3); double asterisk (**) indicates isolation following expression in BL21(DE3)ΔiscR. E289A and E289D were retransformed in BL21(DE3)ΔiscR since the variants had negligible expression yields in BL21(DE3). Note that the BL21(DE3)ΔiscR-expressed variants yielded apo-enzymes with the expected Fe content already in their as purified form and were not semi-enzymatically reconstituted further (N/A). The indicated errors are estimated from the Fe assays, and thus, do not reflect uncertainties in protein concentration as determined by Bradford assays.

Table S2. Specific activities by the holo-*Tam*HydS variants

	**H_2 oxidation**	**H_2 evolution**
	**Benzyl Viologen, –359 mV**	**Methyl Viologen, -446 mV**
**WT**	1.95 ± 0.27	0.09 ± 0.01
**E252V**	nd	0.01 ± 0.01
**E252D**	nd	0.02 ± 0.01
**E289A**	0.04 ± 0.01	0.03 ± 0.01
**E289D**	0.04 ± 0.01	0.06 ± 0.01

Table S2. Tabulated specific activities of *Tam*HydS **WT** and variants in U/mg where one unit (U) of activity catalyzes 1 μmol of H_2 oxidized or produced per min under the utilized assay conditions, which are described in the experimental procedure section. Negligible H_2-oxidation activities that were below the detection limit are indicated as nd–not detected. Error bars indicate standard error, with n = 6 (three technical repeats each for two biological samples). The reduction potentials of BV and MV are indicated in mV versus SHE.

Figure S5. FTIR spectra of *Tam*HydS variants prepared with excess sodium dithionite and H/D exchange for State 1 and State 2

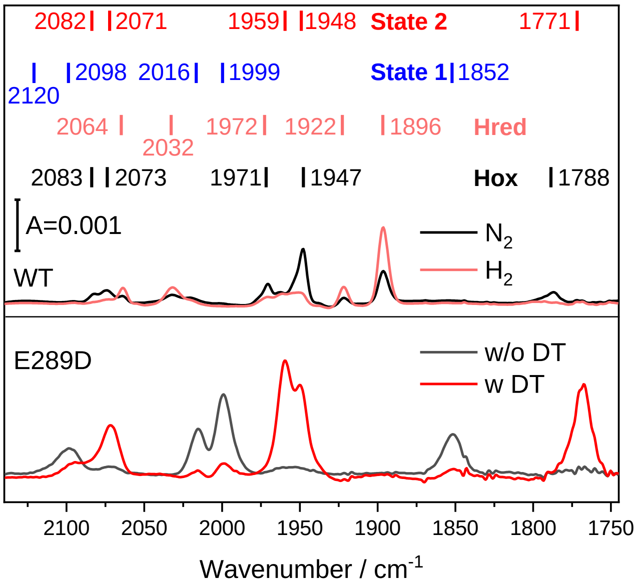

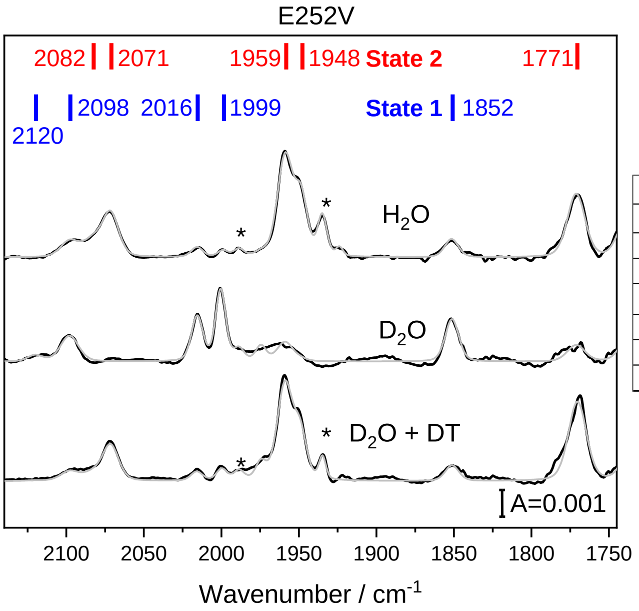

	D_2O	H_2O	
	Bandposition		delta cm^{-1}
State 1	2015,6	2015,9	-0,3
State 1	2000,5	1999,0	1,5
State 1	1851,2	1851,5	-0,3
State 2	1959,0	1958,8	0,2
State 2	1949,0	1948,4	0,6
State 2	1770,3	1771,2	-0,8

Figure S5. FTIR spectra of *Tam*HydS variants prepared with excess sodium dithionite and H/D exchange for State 1 and State 2. (top) FTIR spectra of E289D mildly oxidised by exposure to air (black spectrum, w/o DT) and subsequent exposure to 10 mM sodium dithionite (red spectrum, w DT). (bottom) FTIR spectra of E252V in H_2O, in D_2O (**State 1** accumulated via limited exposure to air) and in D_2O in the presence of additional sodium dithionite (DT); spectra shown in black, fits overlaid (gray lines). No shift larger than 1.5 cm^{-1} could be detected (compare table on the right with fitted CO ligand band positions) that would be indicative of e.g. hydride binding. Peaks of unassigned redox species are labelled with *. None of the variants adopt H_{ox} and H_{red}. Conversely, wild type *Tam*HydS (WT) display exclusively these two latter states (compare top spectra of the top panel).

Figure S6. EPR spectra of holo-*Tam*HydS E252V and E289D variants after treatment with D_2, with and without D_2O.

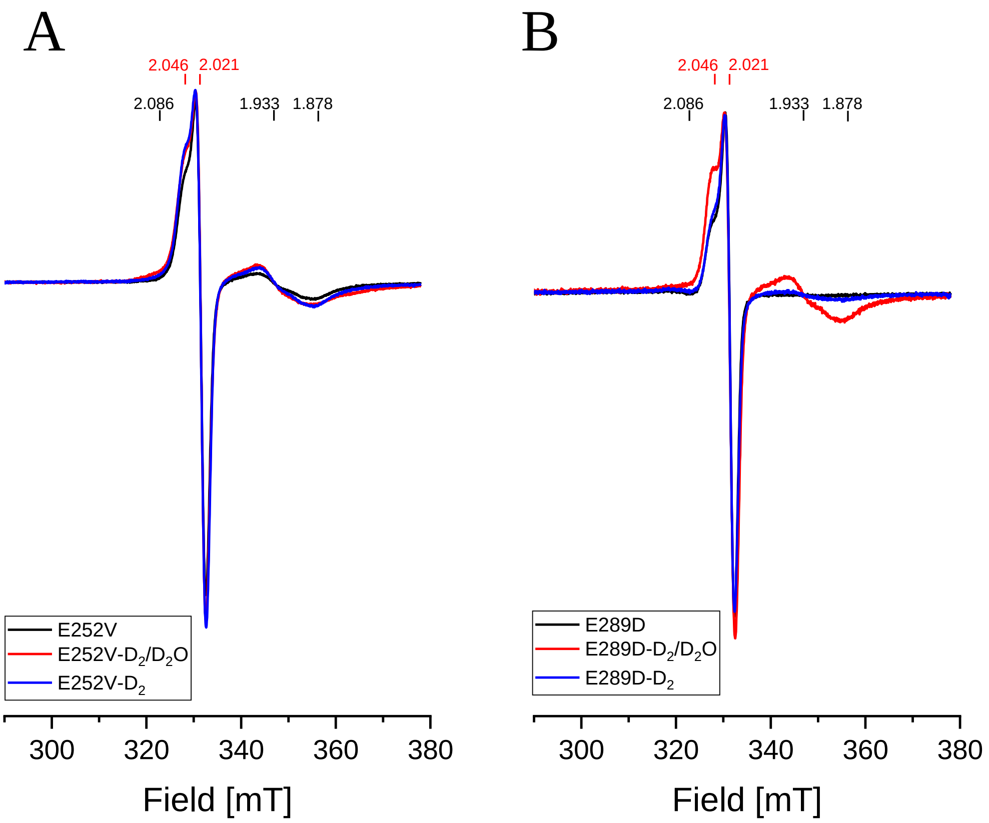

Figure S6. EPR spectra of "as-isolated" and D_2-treated E252V and E289D, with and without D_2O, recorded at 10 K. (A) Comparison of (black) "as-isolated" E252V, (blue) D_2-flushed E252V in Tris buffer (4 µL of 1 mM enzyme in 10 mM Tris-HCl, pH 8.0 + 76 µL 100 mM Tris-HCl, pH 8.0), and (red) D_2-flushed E252V in 95% D_2O (4 µL of 1 mM enzyme in 10 mM Tris-HCl, pH 8.0 + 76 µL D_2O) (B) Comparison of (black) "as-isolated" E289D, (blue) D_2-flushed E289D in Tris buffer (4 µL of 1 mM enzyme in 10 mM Tris-HCl, pH 8.0 + 76 µL 100 mM Tris-HCl, pH 8.0), and (red) D_2-flushed E289D in 95% D_2O (4 µL of 1 mM enzyme in 10 mM Tris-HCl, pH 8.0 + 76 µL D_2O). The g-values as determined in 100 mM Tris-HCl, pH 8.0 are indicated in the figure as vertical bars: Rhombic $[4Fe\text{-}4S]^+$ components in black[2]; **State 2** components in red. A slight narrowing of the rhombic EPR signal for $[4Fe\text{-}4S]^+$ is observed in D_2/D_2O, with a shift of g_1 (2.086) to slightly lower g-values. Spectra normalized at 330 mT to facilitate comparison of the **State 2** axial signal. Normalization factors: (A) E252V (×1), E252V-D_2/D_2O (× 0.95), E252D-D_2 (× 0.93). (B) E289D (× 1), E289D-D_2/D_2O (× 1.7), E89D-D_2 (× 0.68). Spectra collected at 10 K, microwave power = 80 µW, modulation frequency = 100 kHz; modulation amplitude = 10 G; microwave frequency = 9.4 GHz.

Figure S7. EPR spectra of holo-*Tam*HydS E252V and E289D variants with and without sodium dithionite (NaDT) treatment.

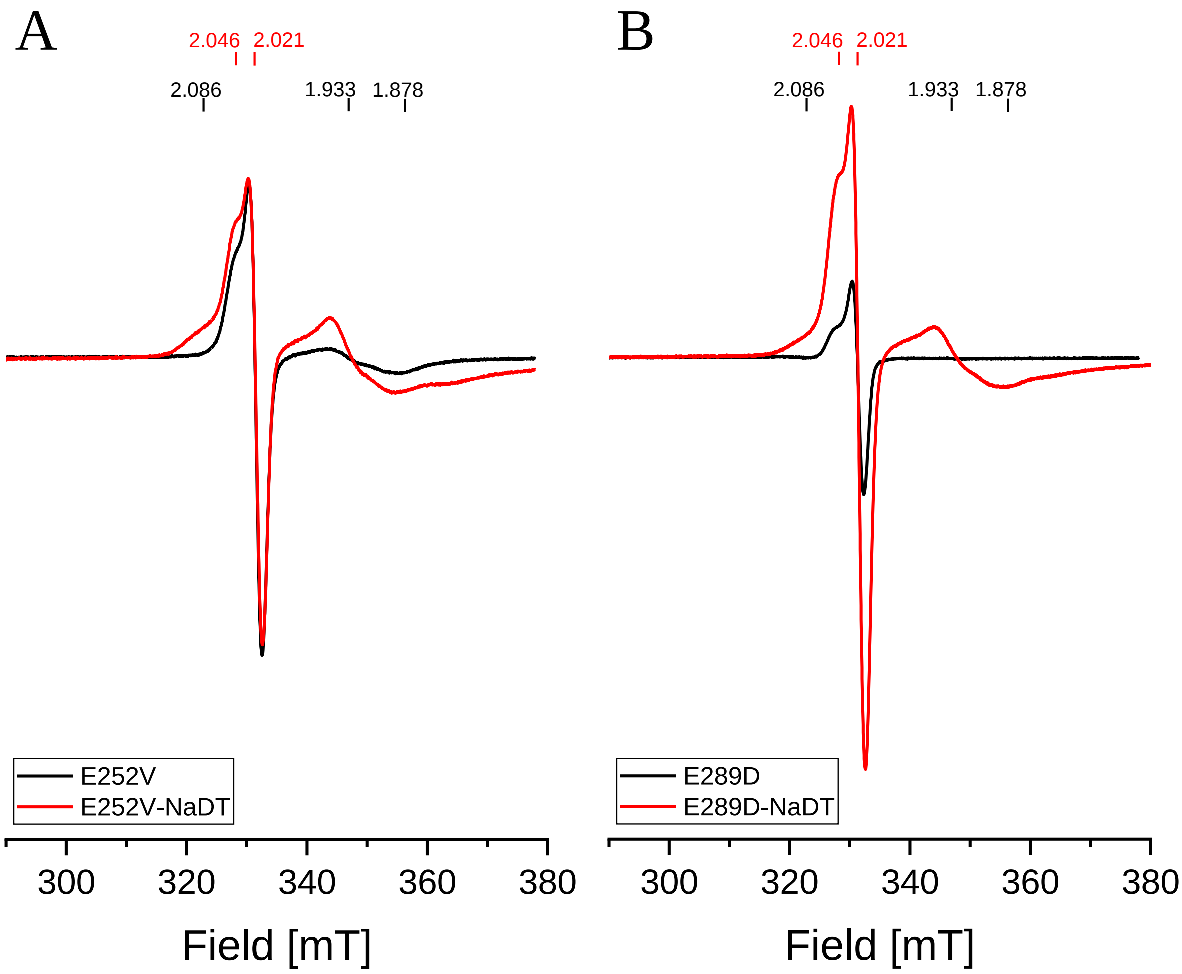

Figure S7. **EPR spectra of "as-isolated" and sodium dithionite-treated E252V and E289D recorded at 10 K.** Comparison of "as-isolated" (black) and sodium dithionite-treated (red) samples of (A) E252V and (B) E289D. The g-values are indicated in the figure as vertical bars: Rhombic $[4Fe\text{-}4S]^{+}$ components in black[2]; **State 2** components in red. For the sodium dithionite-treated samples (NaDT), 1 µL of 100 mM stock solution of NaDT was added into 79 µL of 50 µM enzyme in 100 mM Tris-HCl, pH 8.0, resulting in at least 20-fold excess of NaDT. Spectra collected at 10 K, microwave power = 80 µW, modulation frequency = 100 kHz; modulation amplitude = 10 G; microwave frequency = 9.4 GHz.

Figure S8. FTIR difference spectra of E289A exposed to N_2 and H_2

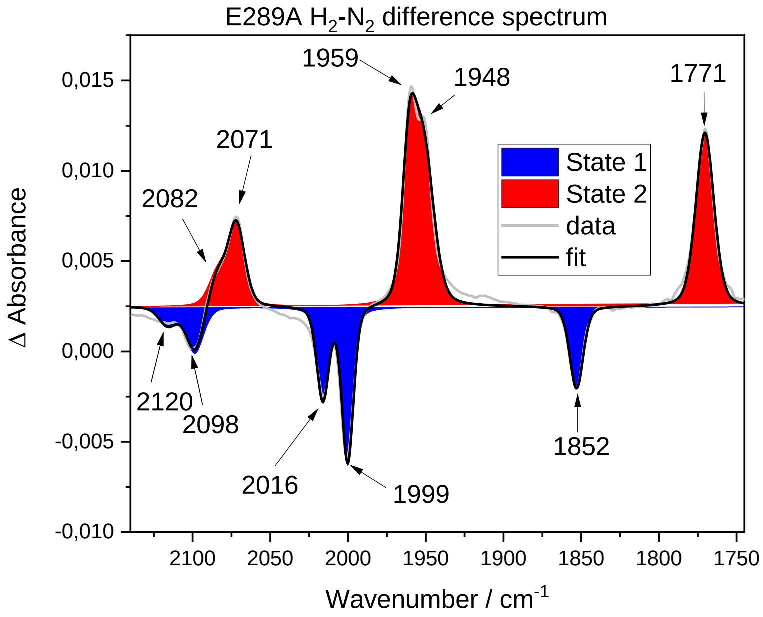

Figure S8. **FTIR difference spectra of E289A exposed to N_2 and H_2.** FTIR difference spectra illustrating the transition from **State 1** (negative blue bands) into **State 2** (positive red bands). **State 1** with bands at 2120, 2098, 2016, 1999 and 1852 cm^{-1} and **State 2** shifted to lower energies (red shifted) with bands at 2082, 2071, 1959, 1948 and 1771 cm^{-1}. Gray line raw data, black line fit.

Figure S9. FTIR difference spectra and redox state population kinetics of *Tam*HydS variant E252V exposed to H_2 and CO

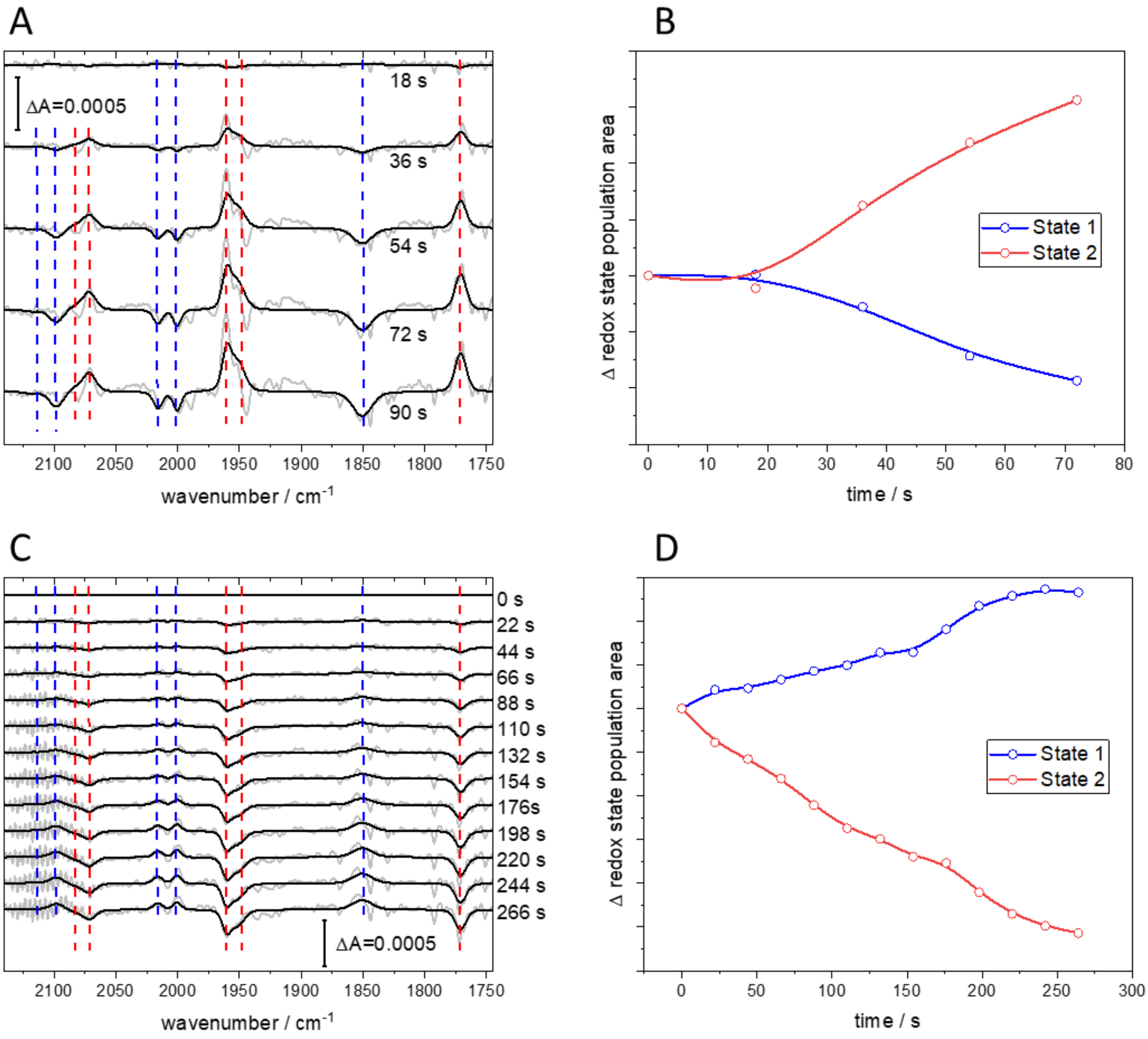

Figure S9. FTIR difference spectra and redox state kinetics of *Tam*HydS variant E252V exposed to H_2 and CO. (A) FTIR difference spectra of E252V exposed to H_2 gas. Within a minute **State 2** (positive bands, red dashed lines) is populated at the expense of **State 1** (negative bands, blue dashed lines). (B) Difference in redox state population plotted over time. Each circle represents the area of IR bands associated with either **State 1** (blue) or **State 2** (red) from Fig. S9A at a given time point. (C) FTIR difference spectra of E252V exposed to CO gas. Within minutes **State 1** (positive bands, blue dashed lines) is populated at the expense of **State 2** (negative bands, red dashed lines). (D) Difference in redox state population plotted over time. Each circle represents the area of IR bands associated with either **State 1** (blue) or **State 2** (red) from Fig. S9C at a given time point.

Figure S10. Experimental versus simulated EPR spectra of **State 2**

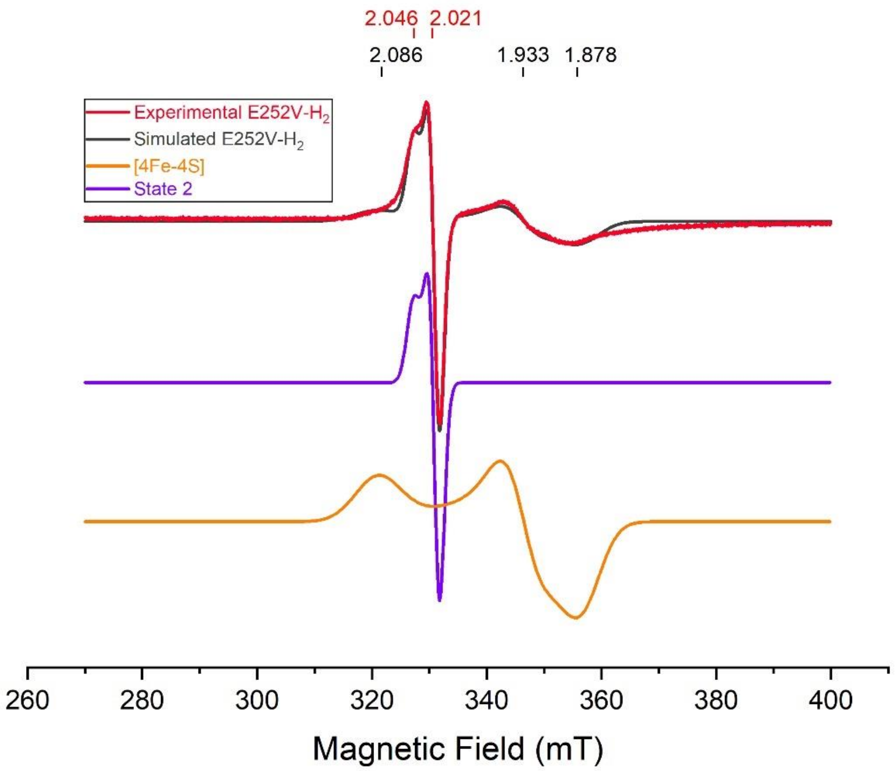

Figure S10. **Experimental and simulated EPR spectra of H_2-flushed E252V.** Comparison of spectrum recorded at 10 K of H_2-flushed E252V (red) and simulated spectrum (black). The two individual components contributing to the overall simulated spectrum are shown in purple (**State 2**, $g_{1,2,3}$ = 2.046, 2.0225, 2.0196; or pseudo axial $g_{\parallel}$ = 2.046, $g_{\perp}$= 2.021 (average)) and orange ($[4Fe4S]^+$ F-cluster, $g_{1,2,3}$ = 2.086, 1.933, 1.878) The g-values are indicated in the figure on vertical bars: Rhombic $[4Fe\text{-}4S]^+$ in black; **State 2** in red. The H_2 flushed sample contained 50 µM of enzyme in 100 mM Tris-HCl, pH 8.0, flushed with H_2 for an hour prior to freezing. Spectrum collected at 10 K, microwave power = 80 µW, modulation frequency = 100 kHz; modulation amplitude = 10 G; microwave frequency = 9.4 GHz. This **State 2** EPR signal has been recognised, though not identified, in WT *Tam*HydS preparations reported earlier (ref 2), in both as isolated (denoted as A1 in Fig. 4A,) and after H_2 treatment (Fig. 4B). H_2 treatment favours the formation of **State 2**, as evidenced in both EPR and FTIR, with parallel accumulation of reduced F-cluster signal(s). For an accurate fitting both components were included in the fitting process.

Figure S11. EPR spectra visualizing the $[4Fe\text{-}4S]^+$ clusters present in H_2-reduced samples of the holo-*Tam*HydS E252V variant.

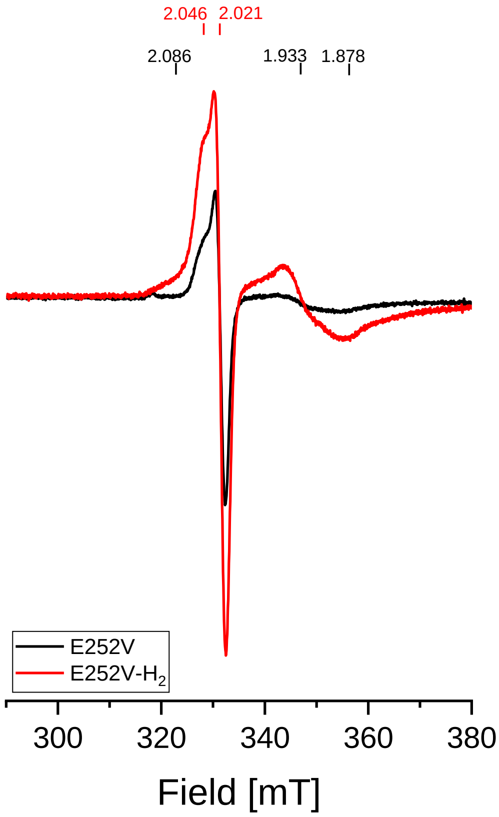

Figure S11. EPR spectra of "as-isolated" and H_2-flushed E252V, recorded at 10 K. Comparison of "as-isolated" (black) and H_2-flushed (red) E252V. The g-values are indicated in the figure on vertical bars: Rhombic $[4Fe\text{-}4S]^+$ components in black[2]; **State 2** components in red. The EPR samples each consist of 50 µM of enzyme in 100 mM Tris-HCl, pH 8.0. The sample reduced with H_2 was prepared by flushing the enzyme solution with H_2 for an hour prior to freezing. Spectra collected at 10 K, microwave power = 80 µW, modulation frequency = 100 kHz; modulation amplitude = 10 G; microwave frequency = 9.4 GHz.

Table S3. Tabulated g-values of the H-cluster of holo-*Tam*HydS WT and E252V and E289D variants.

[FeFe] hydrogenase	Species	g-values			Reference
		g₁	***g₂***	***g₃***	
***Tam*HydS**	H_{ox}-R1	2.109	2.053	2.010	Land et al., 2020[2]
	H_{ox}-R2	2.099	2.044	2.010	
	H_{ox}-CO*	2.034	2.023	2.023	
	A1*	2.041	2.022	2.022	
**E252V variant**	State 2	2.046	2.0225	2.0196	This work
**E289D variant**	State 2	2.046	2.0225	2.0196	This work

Table S3. Tabulated g-values of holo-*Tam*HydS WT, E252V and E289D variants. The asterisk (*) indicates correction of assigned species to the previously reported g-values based on the results of this study. The State A1 in Land et al., 2020[2] most likely corresponds to **State 2** of E252V and E289D variants.

Figure S12. Models for the catalytic cycle of [FeFe] hydrogenases

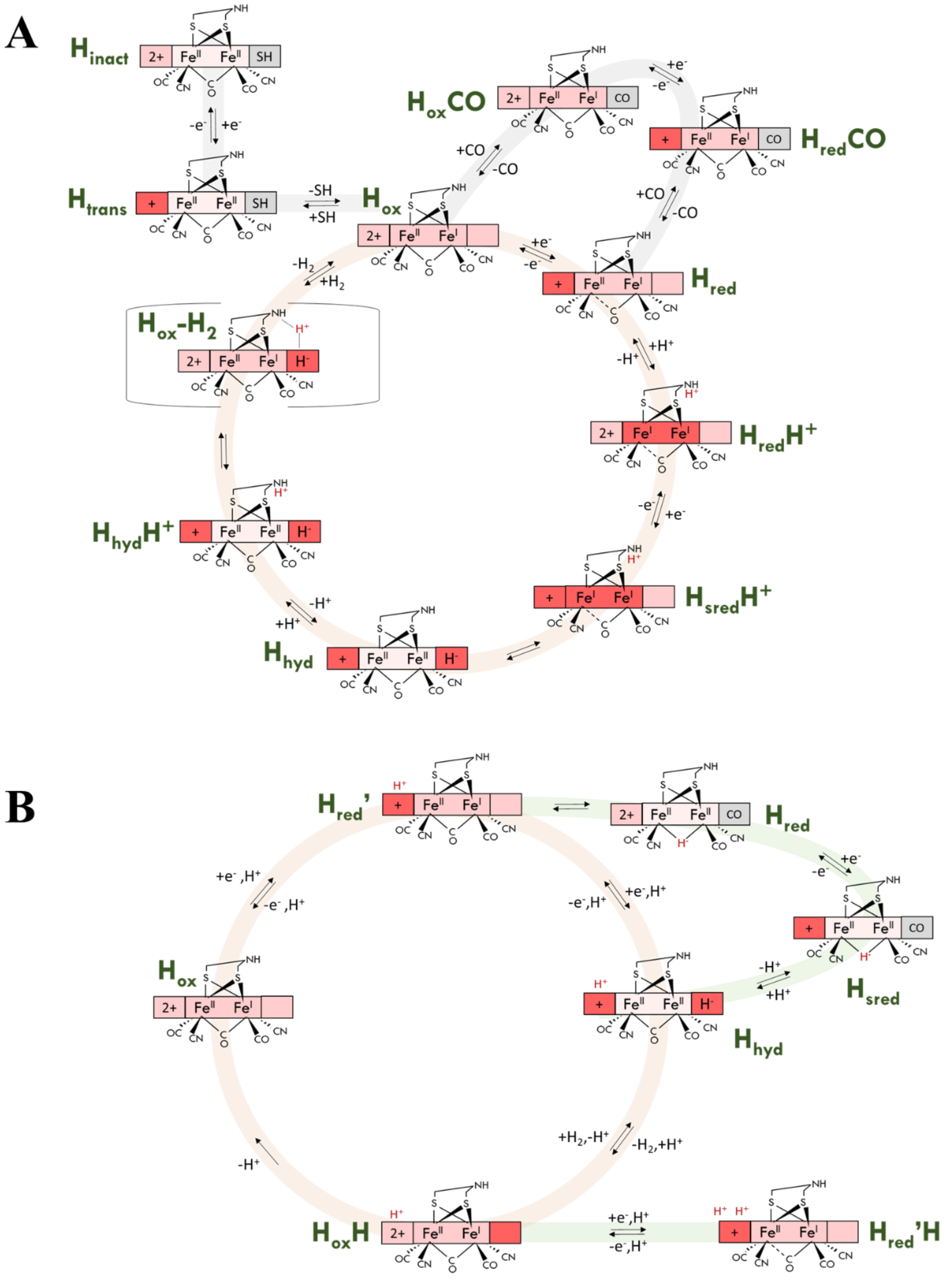

Figure S12. **Schematic representation of the two models for the catalytic cycle of [FeFe] hydrogenase.** The main active pathway is shown as an orange cycle in Model A and B. The charges of the [4Fe-4S] cluster and diiron site of the H-cluster are shown in left and center rectangles, respectively, with varying red intensities depending on the redox state. The rightmost rectangle reflects the ligand of the "open" coordination site of the distal iron. The gray pathways indicate inactivation pathways by thiolates or CO in Model A while the green pathways form the slow catalytic pathway in Model B. Figure adapted from Birrell et al., 2021.[3]

References:

(1) Esselborn, J.; Muraki, N.; Klein, K.; Engelbrecht, V.; Metzler-Nolte, N.; Apfel, U. P.; Hofmann, E.; Kurisu, G.; Happe, T. A structural view of synthetic cofactor integration into [FeFe]-hydrogenases. *Chem. Sci.* **2016**, *7* (2), 959-968.

(2) Land, H.; Sekretareva, A.; Huang, P.; Redman, H. J.; Németh, B.; Polidori, N.; Mészáros, L. S.; Senger, M.; Stripp, S. T.; Berggren, G. Characterization of a putative sensory [FeFe]-hydrogenase provides new insight into the role of the active site architecture. *Chem. Sci.* **2020**, *11* (47), 12789-12801.

(3) Birrell, J. A.; Rodríguez-Maciá, P.; Reijerse, E. J.; Martini, M. A.; Lubitz, W. The catalytic cycle of [FeFe] hydrogenase: A tale of two sites. *Coord. Chem. Rev.* **2021**, *449*, 214191.
